# Genomic Regions Associated with Spontaneous Abortion in Holstein Heifers

**DOI:** 10.3390/genes15121498

**Published:** 2024-11-22

**Authors:** Emaly M. Suarez, Victoria C. Kelson, Jennifer N. Kiser, Kimberly M. Davenport, Brenda M. Murdoch, Holly L. Neibergs

**Affiliations:** 1 Department of Animal Sciences, Washington State University, Pullman, WA 99164, USA; emaly.suarez@wsu.edu (E.M.S.);; 2 Washington Animal Disease Diagnostic Laboratory, Washington State University, Pullman, WA 99164, USA; 3 Department of Animal, Veterinary and Food Sciences, University of Idaho, Moscow, ID 83844, USA

**Keywords:** artificial insemination, cattle, dairy, embryo transfer, genomic selection, loci, spontaneous abortion

## Abstract

**Background/Objectives:** The dairy industry relies on reproductive efficiency to maintain efficient milk production. Spontaneous abortion (SA), defined as pregnancy loss between gestation days 42 and 260, occurred in 4.5% of the artificially inseminated (AI) Holstein heifers and 31.6% of the embryo transfer (ET) recipient Holstein heifers that received in vitro-produced frozen embryos on a single dairy farm in Idaho. **Methods:** A genome-wide association analysis (GWAA) was performed to identify the associations (FDR *p* < 0.05) with SA in heifers that were bred by AI (1351 controls that delivered at term and 63 cases that aborted) that conceived following the first insemination, as well as in 59 controls and 273 cases of ET recipient heifers pregnant from the first ET. **Results:** There were 216 loci and 413 positional candidate genes associated (FDR *p* < 0.05) with SA in the heifers bred by AI in a recessive model and no loci associated with SA in the ET recipients. **Conclusions:** The identification of loci associated with SA in the heifers bred by AI may be used to reduce fetal loss through genomic selection.

## 1. Introduction

The definition of spontaneous abortion (SA), also called fetal loss, is based on developmental changes that occur from fertilization to the differentiation stage that marks the end of the embryonic period at approximately 42 days after insemination, as well as the fetal period that begins after day 42 [1,2,3]. A full-term pregnancy is defined as parturition at or after gestational day 260, when the calf can survive outside the uterine environment [3].

Reproductive efficiency is an important economic trait to the dairy industry. Fertilization rates after one insemination are approximately 90%; however, the percentage of those calving at term is approximately 55% to 60% in heifers [4]. Previous studies have estimated SA in artificially inseminated (AI) heifers to range between 2 and 12%, although some herds have SA rates that exceed 14% [5,6]. Fetal loss is economically unfavorable at any time but is particularly deleterious late in gestation due to increases in the cost of medical care and feed, the number of services needed for a viable pregnancy, increases in the potential number of replacement animals needed due to culling, and a decrease in milk production [7]. Additionally, SA often results in an increased risk for the retention of fetal membranes and the development of endometritis, further reducing reproductive performance while increasing veterinary and labor costs [5]. Based on the net present value in US dollars, De Vries and colleagues [8] estimated an average cost of $855 per incidence of SA, whereas Lee and Kim [9] estimated an overall economic loss from pregnancy loss to be $3523 per cow due to an extended calving interval and increased culling rate [10]. Another study byBellows and colleagues [11] estimated the total yearly net present value cost of female infertility in the dairy industry due to abortions or stillbirths, dystocia, retained placentas, and metritis or pyometra to range from $856 to $876 million annually in US dollars [10].

To reduce the economic burden of pregnancy loss, producers have been selecting for fertility traits such as conception rate (the percentage of inseminated females that become pregnant at each service) and daughter pregnancy rate (percentage of nonpregnant cows that become pregnant during each 21-day period) [12]. Utilizing fertility traits in genomic selection has accelerated the genetic progress of fertility; however, there is a gap in knowledge about why SA occurs and how it can be reduced. The utilization of different breeding systems, such as AI and embryo transfer (ET), adds an additional layer of complexity to SA. This study aims to identify the genomic regions and genes associated with SA in the heifers bred by AI or serving as ET recipients to improve the knowledge of the genomic and physiological mechanisms responsible for SA. These results can be used to identify the genetic differences and similarities resulting in SA for heifers pregnant by AI and those that were recipients of ET.

## 2. Materials and Methods

### 2.1. Study Population and Phenotypes

Nulliparous Holstein heifers (n = 5750) from a dairy farm in Idaho were analyzed for an association with SA. Health and breeding records for heifers were obtained using Dairy Comp 305 (Valley Agricultural Software, Tulare, CA, USA) to identify animals who experienced SA after being bred by AI (n = 63) or as ET recipients (n = 273). Heifers were bred at the observed estrus (n = 1414) or at a timed interval post-synchronization (n = 864). There was no observed difference in abortion rates between the heifers that were bred at the observed estrus or those bred after a double ovulation synchronization protocol was used for the timing of the AI service and ET. Pregnancy was determined using a trans-rectal ultrasound at approximately 30 days post-breeding or transfer. Only heifers who were pregnant from the first AI (n = 1414) or ET (n = 864) were included in the study to eliminate the possible confounding of phenotypes for embryonic loss. Phenotypes were based on whether a heifer maintained a pregnancy to term (control; >260 days of gestation) or experienced SA. Heifers were evaluated and removed from the study if they experienced mastitis, metritis, metabolic issues, lameness, or respiratory disease any time throughout pregnancy. No heifers bred by AI and only one ET recipient heifer was identified as having a health event during gestation. There were 1351 controls and 63 SA heifers bred by AI and 591 controls and 273 SA ET recipient heifers in the study.

### 2.2. Genotyping and Imputation

All animals were genotyped by Zoetis (Parsnippany, NJ, USA) using CLARIFIDE^®^ PLUS. These genotypes were imputed to a higher density (Illumina Bovine HD BeadChip, San Diego, CA, USA), utilizing Beagle (v. 4.1) [13]) and a Holstein reference population of 4800. The 636,042 imputed SNPs had a call accuracy of greater than 95%. This was validated through removing the genotypes from animals in the reference population to approximately 50,000 genotyped SNPs. Genotypes were then imputed up to the BovineHD BeadChip level to calculate the accuracy of the imputation.

### 2.3. Quality Control

Prior to the genome-wide association analysis (GWAA), the imputed genotypes were passed through quality filters. Single nucleotide polymorphisms (SNPs) were removed if they had a call rate < 0.90, had a minor allele frequency (MAF) < 0.01 or that failed (*p* < 1 × 10^−100^) Hardy–Weinberg equilibrium testing. For heifers bred by AI, 22,739 SNPs were removed for call rate, 112,546 SNPs for MAF, and 2649 SNPs for failing Hardy–Weinberg equilibrium testing. Heifers receiving ET had 23,021 SNPs removed for call rate, 97,787 SNPs for MAF < 0.01, and 1295 SNPs for failing Hardy–Weinberg equilibrium testing. Heifers were also subjected to quality control for genotyping call rate (<0.90) and for differences between genotypic and phenotypic sex. No animals were removed for failing quality control. After quality control was complete, 498,036 SNPs for heifers bred by AI and 513,867 SNPs for heifers receiving ET remained for analysis.

### 2.4. Genome-Wide Association Analysis

A GWAA was performed to identify the loci associated with SA in heifers bred by AI or that were ET recipients using the SNP and Variation Suite (SVS) software version 8.1 (Golden Helix, Bozeman, MT, USA). For the GWAA, an efficient mixed-model associated eXpedited (EMMAX) statistical approach using an identity-by-state matrix was used [14]. The general mixed model for EMMAX is expressed as γ=Xβ+Zμ+ε, where γ = the vector of observed phenotypic values, X = a matrix of fixed effects, *β* = regression coefficients, Z = a matrix containing the observed random effects, μ = vector of random effects concerning variants of allele substitutions in the population, and ε = residual effects [14]. Due to the unknown inheritance of SA in cattle, three inheritance models (additive, dominant, and recessive) were analyzed for the AI and ET heifers using SVS.

A principal component analysis was performed to evaluate population stratification. Two distinct clusters within the population were identified based on birth year (Supplemental Figure S1). However, there was no effect of birth year on SA in heifers bred by AI (*p* = 0.053, ANOVA) or that were ET recipients (*p* = 0.053, ANOVA). In addition to birth year, season of conception and service sire were tested as confounding factors for SA. The season that the heifers conceived did not differ (*p* = 0.278, ANOVA) for SA for heifers bred by AI or for heifers that were ET recipients (*p* = 0.096, ANOVA). Spontaneous abortion did differ (*p* = 2.32 × 10^−5^, ANOVA) by service sire for heifers bred by AI and for heifers that were ET recipients (*p* = 4.28 × 10^−7^, ANOVA) and was used as a covariate for all analyses. There was no difference in spontaneous abortion based on AI breeding technicians (*p* = 0.056, ANOVA) or for ET recipient technicians (*p* = 0.486, ANOVA). There was also no difference (*p* = 0.089, ANOVA) in the rate of spontaneous abortion between animals that were bred at the observed estrus or were synchronized. The genomic inflation factor lambda (*λ*_GC_) was calculated in SVS for each inheritance model to identify any potential biases present within the association results [15].

To correct for multiple testing, a false discovery rate (FDR) was calculated in SVS. Associations with SA were identified when FDR < 0.05 [16,17]. When multiple SNPs were associated with SA on a chromosome, the loci were defined by a threshold of D′ > 0.7 as previously described [18,19]. To identify the number of loci associated with SA, a locus consisted of all the SNPs with a D′ > 0.07. For each locus, positional candidate genes were identified based on if they were located within a haplotype of an associated SNP. A haplotype analysis was performed in SVS utilizing the method previously described by Gabriel and colleagues [20] to calculate the average haplotype size for this population, which was 30.5 Kb. Therefore, genes within 30.5 kb 5′ or 3′ to the associated SNP based on the bovine ARS-UCD 1.2 genome assembly [21] were identified as positional candidate genes.

The proportion of variance explained by an SNP was calculated in SVS using the notation of Further Optimization When Covariates Are Present [22,23]. As SNPs within a locus are not independent due to linkage disequilibrium, the sum of the proportion of variance explained for all SNPs exceeds 100%. Relative risk, a measure of the strength of the association between having the unfavorable allele (the allele most common in the cases) and having an SA was calculated utilizing the following equation: a(a + b)/c(c + d), where a is the minor allele frequency in cases, b is the minor allele frequency in controls, c is the major allele frequency in cases, and d is the major allele frequency in controls. The relative risk was calculated for the most significant SNP in each locus. To identify the average relative risk for associated loci, the relative risk for the most significant SNP at each locus was averaged.

To estimate the heritability of SA, a genomic best linear unbiased predictor (GBLUP) analysis [24,25] with the average information algorithm (AI-REML) was used. This method was utilized as the pseudo-heritability estimated by EMMAX can be over-inflated in limited sample sizes.

## 3. Results

**Figure 1:**
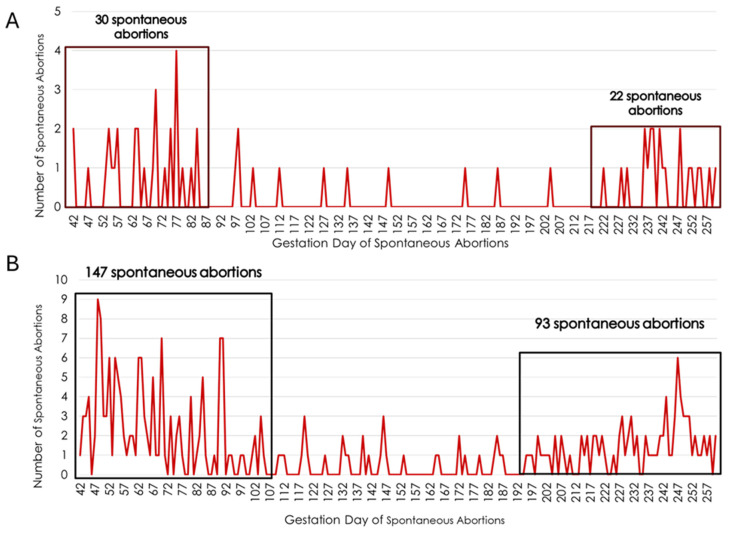
Timing of spontaneous abortions as measured by day the heifer returned to estrus during the fetal period (days 42 to 260 of gestation) for Holstein heifers bred by artificial insemination (panel **A**) or those that were ET recipients (panel **B**). The x-axis lists the gestation day while the y-axis indicates the number of heifers returning to estrus due to a spontaneous abortions occurring on each day of gestation during the fetal period.

The combined AI and ET incidence for SA for first-service Holstein heifers was 14.7%. Heifers bred by AI experienced an SA incidence of 4.2%, while Holstein heifers that were ET recipients experienced an SA incidence of 31.5% for the frozen in vitro-produced embryos. Heifers that spontaneously aborted were more likely to do so during the first trimester in the AI-bred as well as ET recipient heifers (Figure 1). Approximately half of the heifers bred by AI that aborted returned to estrus between gestational days 42 and 85, while approximately 35% of the AI-bred heifers that aborted returned to estrus after day 220 of gestation. Similarly, 54% of the ET recipient heifers that aborted returned to estrus between days 42 and 105, while approximately 34% of ET recipient heifers that aborted returned to estrus due to an SA after day 195 of gestation.

### 3.1. Spontaneous Abortion in AI-Bred Heifers

The *λ*_GC_ for SA in the GWAA of the AI heifers was 0.98, 0.99, and 0.92 for the additive, dominant, and recessive inheritance models, respectively.

**Figure 2:**
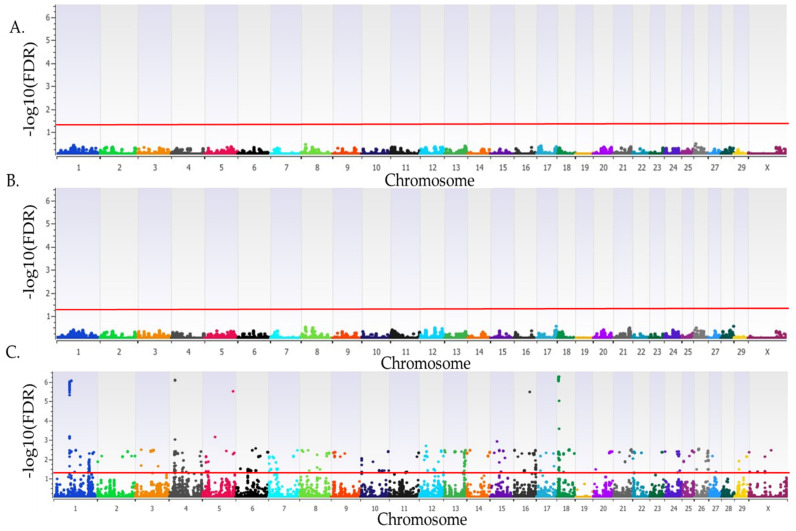
Loci associated with SA in Holstein heifers bred by artificial insemination. Loci for additive (**A**), dominant (**B**), and recessive (**C**) inheritance models. All plots have the *Bos taurus* chromosome on the x-axis, and the −log10(FDR) on the y-axis. Chromosomes are represented by different colors. A red line indicates the threshold for an association (FDR *p* < 0.05). Due to all animals being female the Y chromosome is not present.

There were 216 loci, defined by 890 SNPs, that were associated with SA in AI-bred heifers in the recessive inheritance model (Figure 2; Supplementary Table S1). Twelve loci on BTA1 (three adjacent loci), BTA4, BTA5, BTA16, and BTA18 (six adjacent loci) were characterized by SNPs, with a proportion of variation explained that exceeded 3% with FDR *p* < 4.9 × 10^−6^. The average relative risk for SA for these twelve loci was 2.22. Fifteen additional SNPs that represented an additional four loci on BTA5, BTA12, BTA15, and BTA18 had a proportion of variation explained for SA that exceeded 2%, with an FDR *p* < 0.0021. For all 216 associated loci, the average relative risk for SA was 1.92. There were no loci associated with SA in the additive or dominant inheritance models.

Of the loci associated with SA in the AI-bred heifers in the recessive model, 175 loci (81%) contained positional candidate genes (Supplementary Table S1). The 413 positional candidate genes were grouped by function into 28 groups (Supplemental Table S2). There were 9 positional candidate genes that were previously identified as associated with SA, 152 positional candidate genes previously identified as associated with a fertility trait, and 160 positional candidate genes previously identified as having a role in fertility. Loci that were characterized by the greatest proportion of genetic variance explained for fetal loss included the positional candidate genes:: cilia and flagella associated protein 44 (*CFAP44*), SID1 transmembrane family member 1 (*SIDT1*), spindle and centriole associated protein 1 (*SPICE1*), phospholipase A1 member A, (*PLA1A*), CD80 molecule (*CD80*), ADP-ribosylarginine hydrolase (*ADPRH*), MAF bZIP transcription factor (*MAF*), WW domain containing oxidoreductase (*WWOX*), vesicle amine transport 1 like (*VAT1L*), ENTH domain-containing 1 (*ENTHD1*), GRB2 related adaptor protein 2 (*GRAP2*), constitutive photomorphogenic 1 (*COP1*), *LOC101902700*, and *LOC112442252*.

The estimated heritability for SA in heifers bred by AI was 0 (±0.029). This estimated heritability could reflect that the associations with SA were in the recessive inheritance model. As heritability estimates are the measures of additive genetic variance, loci associated with SA that are identified in the recessive inheritance model would not contribute to this estimate.

### 3.2. Spontaneous Abortion in Heifers That Were ET Recipients

**Figure 3:**
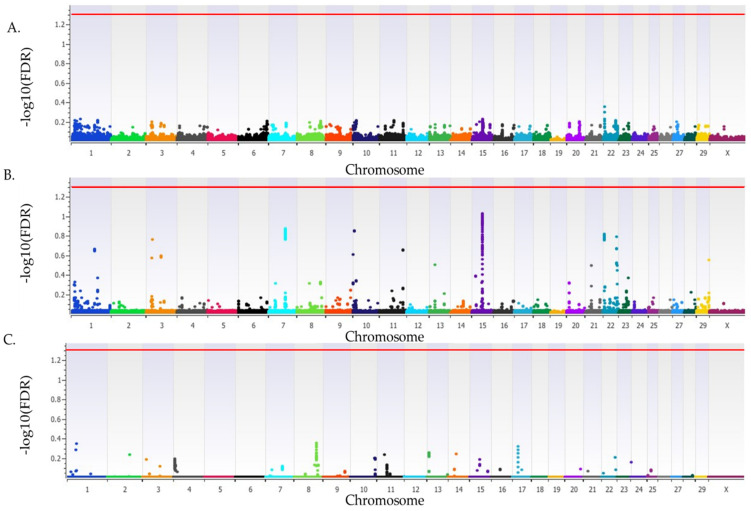
Loci associated with SA in Holstein heifers that were embryo transfer recipients. Loci for additive (**A**), dominant (**B**), and recessive (**C**) inheritance models. All plots have the *Bos taurus* chromosome on the x-axis, along with the −log10(FDR) on the y-axis. Chromosomes are represented by different colors. A red line indicates the threshold for an association (FDR *p* < 0.05). Due to all animals being female the Y chromosome is not present.

The *λ*_GC_ for SA for heifers that were ET recipients was 1.01, 1.01, and 1.01 for the additive, dominant, and recessive inheritance models, respectively. There were no loci that were associated (FDR *p* < 0.05) with SA for ET recipient heifers in the additive, dominant, or recessive inheritance models (Figure 3). The estimated heritability of SA in ET recipient heifers was 0.11 (±0.07).

## 4. Discussion

The frequency of SA in the Holstein heifers bred by AI was 4.2%, which is consistent with other reports where the rate of SA ranged from 2 to 12% [5,26]. The frequency of SA in the Holstein heifers that received in vitro frozen embryos was higher (31.5%) than those bred by AI, as well as higher than studies that transferred freshly collected embryos which reported a 10 to 13% incidence of SA [27]. Whether this large increase in SA was due primarily to the embryos being frozen and thawed compared to freshly fertilized unfrozen embryos in the Hasler [27] study is unknown. Other factors that could have contributed to pregnancy loss in the ET recipients include the site of embryo deposit in the uterus, the developmental stage and quality of the embryo, infectious disease, and the maternal and paternal genetics of those used to produce the embryos. When comparing the SA frequency of the heifers with primiparous cows (those that have previously calved once) on the same dairy farm, the SA rates were higher (9.2%) for the AI-bred cows (679 controls and 69 cases) but lower (14.8%) for the cows that were ET recipients (236 controls and 41 cases). As the heifers and cows for the AI-bred and ET recipients were from the same dairy farm, were managed the same, and had the same exposure to infectious disease, we can only speculate why these incidence rates are different. The identification of loci and positional candidate genes associated with SA in the AI-bred and ET recipients is the first step in investigating the potential causes of these differences.

The average relative risk of the most significant SNP for each of the 216 loci associated with SA was 1.9, indicating that the selection of the undesirable alleles for these loci would almost double the risk of an SA in heifers bred by AI (Supplemental Table S3). All 216 loci had a proportion of variation that exceeded 1%. Selecting alleles that are favorable that explain a large amount of the variance associated with SA could be very beneficial to the dairy industry. There is great potential for utilizing these genomic regions to not only decrease the occurrence of SA but also to aid the industry by reducing the economic burden of SA.

Eight (*CFAP44*, *SPICE1*, *ADPRH*, *MAF*, *WWOX*, *VAT1L*, *CD80*, and *PLA1A*) of the positional candidate genes with the highest proportion of variation and highest relative risk for SA have functions that have been identified as relevant to fetal loss. *CFAP44* null male mice are sterile due to cilia and flagella dysfunction [28]. Primary cilia regulate cellular signaling, as well as transduce and sense extracellular stimuli. In women, abnormal ciliogenesis has been indicated as a possible cause of recurrent miscarriages [29]. *SPICE1*, a spindle- and centriole-associated protein is involved in the spindle and centrosome assembly in mitosis and meiosis to aid in chromosome segregation [30,31]. Centrosomes are also implicated in cell migration, cell polarity, and vesicle trafficking [31]. The important role of centrosomes and spindles in mitosis can have a large impact on normal fetal development, with errors leading to the occurrence of SA. *ADPRH* removes mono-ADP-ribose from the arginine residues of proteins [32]. This plays a crucial signaling function in the cell cycle, DNA damage repair, inflammatory response, and response to virus infection [32]. Proper progression of the cell cycle is important for fetal development, as is DNA damage repair in rapidly growing fetuses. If these processes are disrupted, it can lead to a greater risk for SA. Inflammatory responses can play a key role in SA as it is a known risk factor for SA [33]. For example, another one of these genes, *CD80*, plays a role in regulating immune response and its overexpression can interfere with T-cell apoptosis, leading to a rejection of the fetus [33]. *MAF* transcription factors are important for the regulation of development and the differentiation of many organs and tissues [34]. These transcription factors have also been indicated to be involved in the regulation of hormonal systems, especially concerning glucose metabolism [34]. These genes could be impactful to SA as the proper development of organs is crucial for fetal survival. There is also a lot of energy demand for fetal development, so transcription factors involved in glucose metabolism are important for supplying energy for fetal growth. *WWOX* is involved in the regulation of cellular glucose and energy metabolisms or metabolic homeostasis, which is important for fetal growth [35]. The *PLA1A* gene has an important role in controlling the fetal and uteroplacental blood flow necessary for nutrient transport and waste removal to aid fetal growth and development [36,37]. An imbalance or overexpression of *PLA1A* could potentially lead to issues that cause SA. Finally, *VAT1L* has been predicted to be involved in the transport of amines within vesicles in cells [38]. This is most likely to function as an oxidoreductase with potential zinc ion binding activity [38,39]. This gene was differentially expressed between the isthmus of pregnant (multiple embryo model) and cyclic heifers that were assessed using reverse transcription quantitative polymerase chain reactions (RT-qPCR) [38,39].

Of the loci identified in the heifers bred by AI, six loci on BTA9 (13 Mb), BTA16 (69 and 80 Mb), BTA18 (9 Mb), BTA21 (42 Mb), and BTAX (142 Kb) were previously identified as associated with SA in Holstein cattle [26]. These loci contain the positional candidate genes (*CD109*, *CDH13*, *KLHL4*, *NUBPL*, *PTPN14*, and *SYT2*). Five of these genes (*CD109*, *CDH13*, *PTPN14*, *KLHL4,* and *NUBPL*) have functional roles that support their importance in pregnancy and will be further discussed.

*CD109* on BTA9 is a cell surface glycoprotein found on hematopoietic stem and progenitor cells, activated platelets, and T cells [40]. *CD109* is a carrier of antigenic determinant human platelet antigen-15 (HPA-15). In humans, mismatches between maternal and paternal serum human platelet antigen cause alloimmunization and can lead to fetal and neonatal alloimmune thrombocytopenia as a result of the alloantibodies crossing the placenta. The crossing of the alloantibodies often leads to bleeding complications in the fetus and neonate [41]. *CDH13* (placental T-cadherin) on BTA18 plays a role in cell migration, brain development, and transcription repression as a tumor suppressor gene. It is correlated with trophoblastic invasion anomalies that lead to fetal growth restriction in humans, which can lead to fetal demise [42,43]. Protein tyrosine phosphatase non-receptor type 14 (*PTPN14*) on BTA16 is also essential in human trophoblast stem cells in placental development [44]. *PTPN14* is a regulator of trophoblasts [44], which are important for nutrition distribution through the placenta to the fetus. Kelch-like family member 4 (*KLHL4*) on BTAX is associated with fatal and non-fatal disorders of macro/microcephaly, as well as body mass index disorders due to mutations that result in gene expression differences in humans in the nervous system and in cellular metabolism [45]. NUBP iron–sulfur cluster assembly factor, mitochondrial (*NUBPL*) on BTA21 plays a crucial role in complex I assembly of the respiratory complex, with recessive variants in the gene being causative for a rare mitochondrial complex I deficiency disorder [46,47]. Using mice as a model, a lack of NUBPL leads to fetal lethality due to a lack of trophoblast expansion [46,48]. Finally, synaptotagmin 2 (*SYT2*) on BTA16 is the major isoform that is expressed at the neuromuscular junction with dominant missense variants having been reported as a rare cause of distal motor neuropathy and myasthenic syndrome [49]. *SYT2* variants have been associated with reduced fetal movements during pregnancy in women [49]. The *SYT2* protein is found in synaptic vesicle membranes and acts as a calcium sensor during vesicular trafficking and exocytosis. This is important as the placenta releases extracellular vesicles into both the maternal and fetal circulation to carry proteins, lipids, nucleic acid, and other molecules that interact with the maternal tissues to regulate both maternal and fetal circulation [50,51].

Additionally, five loci associated with SA in heifers bred by AI were shared with other fertility phenotypes or fertility indexes. Two of these loci are located on BTA12 at 21 and 23 Mb and were shared with Höglund and colleagues’ [52] association with a fertility index in Nordic Red cattle. This fertility index is comprised of multiple traits, such as the number of inseminations per conception, the length in days of the interval from calving to first insemination, days from first to last insemination, and the 56-day non-return rate in Nordic Red cattle [52]. As this index included the interval between calving and first insemination, which is often lengthened by SA, this study may have indirectly measured SA as one of the fertility traits. SA also tends to increase the number of inseminations to a successful pregnancy, which was another trait included in this index. The locus on BTA12 at 23 Mb shared the same SNP (rs134485572; Supplemental Table S2) with the fertility index and SA; however, the favorable allele for the fertility index was not reported by [52], and so it is not known if the favorable alleles are shared.

The third locus, associated with SA located on BTA16 at 2 Mb was also shared with an association with the 56-day non-return rate in Nordic Holstein, Nordic Red, and Jersey cows [53]. This shared locus may be due to SA having an influence on the 56-day non-return rate as some early SA would result in an animal being recorded as open thus decreasing the 56-day non-return rate.

The fourth locus associated with SA on BTA25 at 25 Mb was also associated with the cow conception rate (CCR), daughter pregnancy rate (DPR), and days open in the Holstein cattle [54,55]. Days open is a phenotype that could be lengthened due to SA, whereas CCR and DPR may have similar physiological pathways as SA. As SA increases the risk for metritis, mastitis, and retained placentas that could potentially increase CCR and DPR. The fifth locus associated with SA on BTA17 at 14 Mb was also associated with DPR [56]. The sharing of two loci associated with SA and DPR suggests that a similar physiological process could be influencing both traits.

When investigating the positional candidate genes associated with SA in heifers bred by AI, 160 positional candidates were previously identified as important for fertility (Table 1). Nine genes (*ASAP1*, *COG5*, *DGKB*, *MASP1*, *PRKG1*, *RBFOX1*, *TDRD9*, *TENM3*, and *TNR*) have previously been identified as positional candidate genes associated with early embryonic loss in Holstein cattle [18,57]. Up to 60 of the 160 genes were also identified as the leading genes for early embryonic loss [18,57]. Additionally, 77 of the positional candidate genes for SA in AI-bred heifers were differentially expressed in the tissues of the heifers based on their fertility status [58], while 80 positional candidate genes were expressed in the reproductive tissues of Holstein cattle based on single-cell data (Table 1) [59]. Notably, 10 (*EGFR*, *FGFR2*, *PDGFA*, *PRKG1*, *PTGFR*, *RASGRP2*, *SLC26A4*, *SMYD2*, *TCF4*, and *UPK1B*) of the 160 total positional candidate genes identified in these studies were shared between embryonic loss and gene expression studies. Nine positional candidate genes (*CD109*, *KLHL4*, *PTPN14*, *CDH13*, *SYT2*, *NUBPL*, *CD80*, *ETV1*, and *PLA1A*) associated with SA were also identified as having a role in fertility in cattle and other species, underscoring the importance of their further investigation to understand the causes of SA and the use of these genomic regions for genomic selection in heifers bred by AI.

Two of these positional candidate genes (*CD80* and *ETV1)* for AI-bred heifers were previously identified as the leading genes in gene set enrichment analyses for embryonic loss [18,57]. *CD80* was an enriched leading gene in heifers bred by AI for embryonic loss, while *ETV1* was a leading gene enriched for embryonic loss in heifers and primiparous cows [18,57]. *CD80* has an important role in regulating immune responses, with the overexpression of the gene interfering with T-cell apoptosis and thus leading to a rejection of the fetus during pregnancy [33]. *ETV1* is a member of the ETS family of transcription factors that modulate cellular processes such as cell growth, angiogenesis, proliferation, migration, and differentiation [60]. These transcription factors are associated with mesenchymal–epithelial interactions and changes in extracellular matrix proteins which contribute to tissue remodeling and integrity [61]. Unique expressions of ETS transcription factors have been found in developing tissues responsible for producing lymphocytes, antibodies, bone marrow, brain and central nervous system, bone, and mammary gland [61]. This indicates an important role of these genes in the development of systems necessary for life.

A third positional candidate gene for one of the most highly associated loci for SA was *PLA1A*. *PLA1A* is differentially expressed in the endometrium of pregnant high fertile and sub fertile beef heifers when compared to their non-pregnant counterparts [58]. There was a higher expression of *PLA1A* in the endometrium of highly fertile and sub-fertile pregnant heifers compared to those who were not pregnant [62]. PLA1A is a phospholipase involved in arachidonic acid metabolism. In women, *PLA1A* plays a significant role in the control of fetal and uteroplacental blood flow and the initiation of parturition [36]. Arachidonic acid is important in fetal growth through its transformation via the cyclooxygenase pathway that initiates the formation of prostaglandins and thromboxane, which are related to fetal growth retardation [63].

There are several Holstein infertility haplotypes (HH) that have been identified and are currently being commercially tested for. These haplotypes include HH1 to HH6 [64] and are located at BTA5 at 63 Mb (HH1), on BTA1 at 94 Mb (HH2), on BTA8 at 95 Mb (HH3), on BTA1 at 1.3 Mb (HH4), on BTA9 at 92 Mb (HH5), and on BTA16 at 29 Mb (HH6) [65,66]. The nearest locus associated with SA and one of the Holstein infertility haplotypes was 161,374 bp away from HH6 on BTA16. The next closest loci identified as associated with SA were −2 to 3 Mb away from HH2 and HH3. Any other loci associated with SA were over 19 Mb away from the other identified Holstein haplotypes, indicating that these are independent regions.

## 5. Conclusions

There were 216 loci and 413 positional candidate genes associated (FDR *p* < 0.05) with SA in the AI-bred Holstein heifers. Six loci were validated for SA in the previous studies, and 160 positional candidate genes can be supported for their role in fertility based on their function and identification in the other fertility-related studies.

The average relative risk for the unfavorable allele associated with SA for the most significant SNP for each of the associated loci was 1.9, demonstrating the value of these loci for predicting SA. Similarly, the variance explained for 16 loci exceeded 2%, with the highest variance explained for a single locus being 3.5% for the locus on BTA5 at 111 Mb. These loci are excellent candidate loci for genomic selection to reduce SA.

No loci were identified as associated with SA in the ET recipient heifers, in contrast to the identification of many loci associated with SA in the heifers bred by AI. Differences between the success of pregnancies initiated by breeding by AI and of heifers serving as recipients of ET have previously been noted, but the genomic foundation of these differences has yet to be established. This study provides the first investigation of loci associated with SA in ET recipient heifers after day 42 of gestation.

Spontaneous abortion in Holstein dairy cattle leads to greater culling rates, a decrease in milk production, and often a greater calving interval. Spontaneous abortion costs producers millions of dollars each year. By identifying the genomic regions associated with SA, especially when utilizing breeding technologies such as ET and AI, there is more potential for using genomic selection to produce animals that are more reproductively efficient.

## Figures and Tables

**Table 1:** Positional candidate genes for spontaneous abortion shared with fertility traits.

Study ^1^	Trait	Positional Candidate Genes ^2^
Moraes et al., 2018 [58]	Differentially expressed genes based on fertility status	***ADIPOR1***, ***ADRA2C***, ***ADTRP***, *AFF2*, ***AKAP6***, ***ANKH***, ***B4GALT4***, *BNIP3*, ***C3H1orf54***, *CAMK1G*, *CAMSAP2*, ***CCDC80***, ***CD109***, *CNTNAP2*, ***COL14A1***, ***CYB5R1***, *CYP2C18*, *CYP2C87*, *DCLK1*, *DDX43*, ***DGKB***, *DNAH5*, ***EGFR***, ***EYA2***, ***FGFR2***, *HEATR5A*, *HHIPL1*, *HS2ST1*, ***IFI27***, *IQCD*, *ISG12(B)*, ***ITM2B***, ***ITPKB***, *LOC100847759*, *LOC101902226*, *LOC101902757*, *LOC101903905*, *LOC101905203*, *LOC104974530*, *LOC516599*, *LOC540627*, *LOC617402*, *LOC783376*, ***MAP1B***, ***MTUS2***, *NBEA*, *NRXN2*, *OLFM2*, *OTULIN*, ***PDGFA***, ***PIK3C2B***, *PLA1A*, ***PRKG1***, *PRRX1*, ***PTGFR***, ***RASA3***, ***RASGRP2***, ***RBFOX1***, ***RBM47***, ***RNF7***, ***SLC26A4***, ***SMIM13***, ***SMYD2***, ***SOX5***, ***SPATA2***, ***SPON1***, ***TCF4***, *TENM3*, *TMEM170B*, *TNR*, *TP53INP2*, *TPCN1*, ***TRIM34***, *TRIM36*, ***UPK1B***, ***WDR12***, ***ZNFX1***
Davenport et al., 2024 [59]	Gene expression in placenta of Bos taurus cattle	***ADTRP***, ***AKAP6***, ***ANKH***, ***AP1M1***, *ARRDC1*, *ATP5MPL*, ***B4GALT4***, ***C3H1orf54***, *CA14*, *CARF*, ***CCDC80***, ***CD109***, ***CD19***, *CDCA5*, *CDYL2*, ***CEP192***, *CMC2*, ***COL4A5***, ***COL4A6***, *CROCC*, ***CYB5R1***, ***DPYSL2***, *ECM1*, ***EGFR***, ***FGFR2***, *GCH1*, *GPR143*, ***HBP1***, ***IFI27***, ***ITM2B***, ***ITPKB***, ***LSM14A***, ***MAP1B***, *MBOAT7*, ***MDM4***, *MGC133636*, *MGMT*, *MPDZ*, ***MTUS2***, ***NECAP2***, *NOC3L*, ***PDGFA***, *PIGU*, *PIN1*, *PLCE1*, *PPP1R15B*, ***PRKG1***, *PRPF31*, *PTGES*, ***PTGFR***, *PTPN14*, ***RABEP2***, ***RASGRP2***, ***RBM47***, *REST*, *RNF130*, ***RNF7***, *SERTAD2*, *SHROOM2*, ***SLC26A4***, ***SMIM13***, ***SMYD2***, ***SOX5***, ***SPATA2***, ***SPON1***, *STIM2*, ***TCF4***, *TMC4*, *TMEM225B*, ***TRIM34***, *UBL3*, ***UCP2***, ***UPK1B***, *WDFY2*, ***WDR12***, *ZMYND19*, *ZMYND8*, *ZNF655*, ***ZNFX1***
Galliou et al., 2020 [18]	Heifer embryonic loss	***ADIPOR1***, ***ADRA2C***, ***AP1M1***, ***ASAP1***, ***ATP2A1***, *CD80*, *CENPN*, ***CEP192***, *CNOT3*, ***COL14A1***, ***COL4A5***, ***COL4A6***, ***DGKB***, ***DPYSL2***, ***ETV1***, *FMO3*, *FUT8*, *GALNT12*, *GORAB*, *GPC6*, *GRAP2*, *GRIK2*, ***HBP1***, *MRPS21*, ***NECAP2***, *PRKG1*, *PTGES*, ***PTGFR***, ***RABEP2***, ***RASA3***, ***RASGRP2***, ***RBFOX1***, ***SMYD2***, *TFPT*, ***UPK1B***
Kiser et al., 2019 [57]	Primiparous embryonic loss	***AP1M1***, *APH1A*, ***ASAP1***, ***ATP2A1***, ***CD19***, *CDH13*, ***COL4A6***, ***DGKB***, ***DPYSL2***, ***EGFR***, ***ETV1***, ***EYA2***, ***FGFR2***, *GAN*, *IL21*, *LAMB3*, ***LSM14A***, ***MDM4***, ***NECAP2***, *NRCAM*, *NUP50*, *OR52H1*, ***PDGFA***, ***PIK3C2B***, ***PRKG1***, ***PTGFR***, *PYGM*, ***RABEP2***, ***RASGRP2***, ***RBFOX1***, *SH2B1*, ***SLC26A4***, *SMAD4*, *SPICE1*, ***TCF4***, ***UCP2***, *UCP3*, ***UPK1B***, *VPS51*

^1^ Citations for previous study that shared fertility positional candidate gene results. ^2^ List of positional candidate genes for SA that were shared with other fertility traits. **Bolded** positional candidate genes are genes that are shared between the previous fertility studies.

## Data Availability

The original contributions presented in this study are included in the article/Supplementary Materials. Further inquiries can be directed to the corresponding author.

## Supplementary Materials

The following supporting information can be downloaded at: https://www.mdpi.com/article/10.3390/genes15121498/s1, Figure S1: Principal component analysis to assess population stratification; Table S1: SNPs associated with SA in heifers bred by AI; Table S2: Genes associated with spontaneous abortion in heifers bred by AI, grouped by function; Table S3: Relative risk for the most significant SNPs representing the 216 loci.
