# Supplementary material for: Genomic Regions Associated with Spontaneous Abortion in Holstein Heifers
Source: Genes (Basel). 2024 Nov 22;15(12):1498. doi: 10.3390/genes15121498 (PMC11675479; doi:10.3390/genes15121498)
Supplement: Supplementary file 1 [file genes-15-01498-s001.zip › genes-3320631-supplementary.pdf]

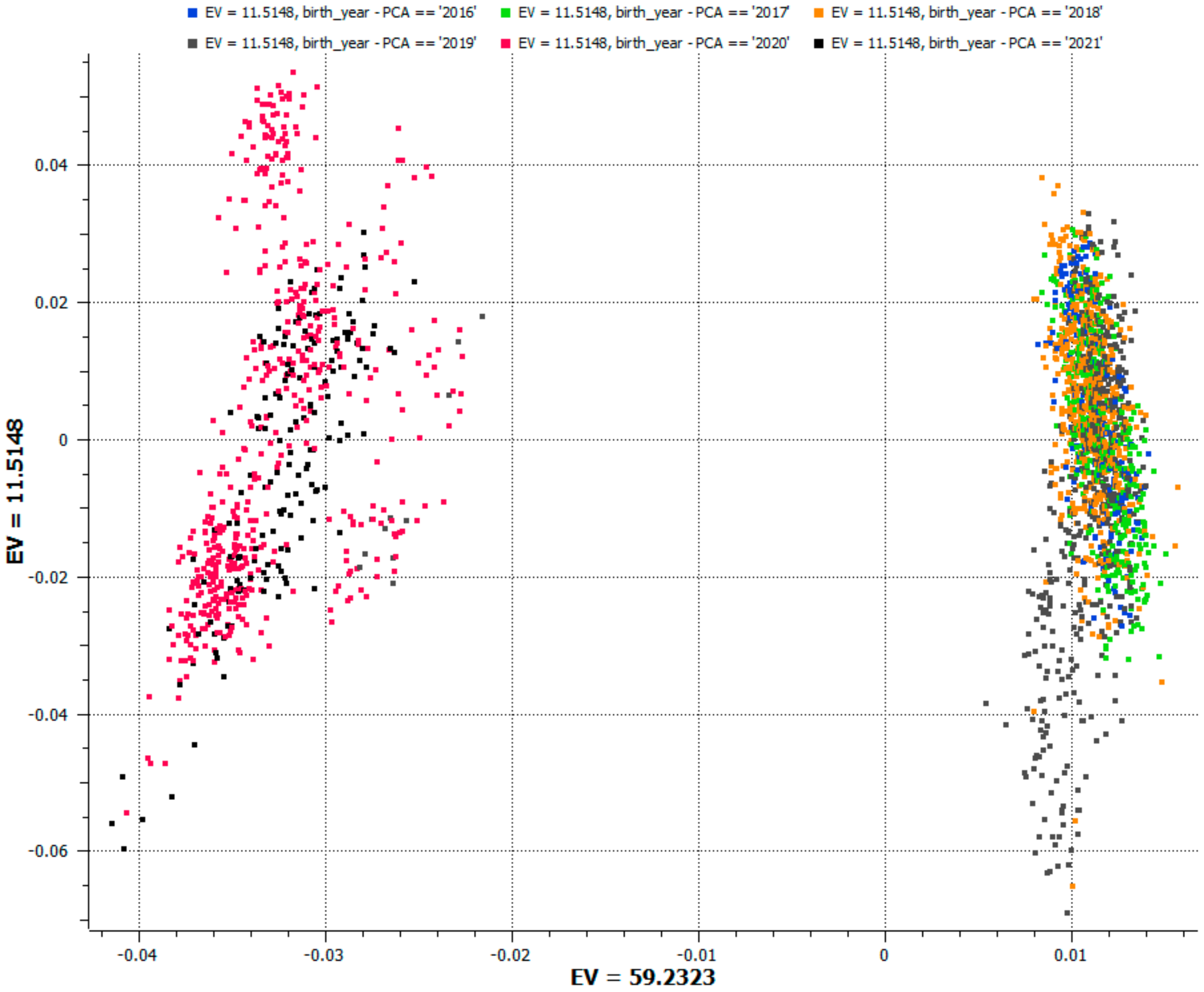

Figure S1: Principal component analysis to assess population stratification of all heifers, colored by birth year.

Table S1: Single nucleotide polymorphisms associated with spontaneous abortion in heifers bred by artificial insemination with the recessive inheritance model.

BTA[1] (locus)	Position (Mb)[2]	FDR[3]	FA Freq[4]	POV[5]	Positional Candidate Gene(s)[6]
1 (1)	57	$7.53x10^{-3}$	0.85	0.015	***CCDC80,*** *LOC112447301*
1 (1)	57	$4.97x10^{-3}$	0.84	0.016	***CCDC80,*** *LOC112447301*
1 (1)	57	$4.96x10^{-3}$	0.84	0.016	***CCDC80,*** *LOC112447301*
1 (1)	57	$4.94x10^{-3}$	0.84	0.016	***CCDC80,*** *LOC112447301*
1 (1)	57	$4.93x10^{-3}$	0.84	0.016	***CCDC80,*** *LOC112447301*
1 (1)	57	$4.92x10^{-3}$	0.84	0.016	***CCDC80,*** *LOC112447301*
1 (1)	57	$3.47x10^{-3}$	0.85	0.017	***CCDC80,*** *LOC112447301*
1 (1)	58	$4.83x10^{-6}$	0.98	0.033	***CFAP44***
1 (1)	58	$8.3x10^{-4}$	0.97	0.022	***CFAP44***
1 (1)	58	$8.15x10^{-4}$	0.97	0.022	***CFAP44***
1 (1)	58	$7.94x10^{-4}$	0.97	0.022	***CFAP44***
1 (1)	58	$3.6 x10^{-6}$	0.98	0.033	***CFAP44***
1 (1)	58	$2.90x10^{-6}$	0.98	0.033	***CFAP44***
1 (1)	58	$2.41x10^{-6}$	0.98	0.033	***CFAP44***
1 (1)	58	$2.07x10^{-6}$	0.98	0.033	***CFAP44***
1 (2)	58	$2.39x10^{-2}$	0.92	0.013	***CFAP44***
1 (2)	58	$7.74x10^{-4}$	0.94	0.022	***CFAP44,*** *SPICE1*
1 (2)	58	$7.55x10^{-4}$	0.97	0.022	***CFAP44,*** *SPICE1*
1 (2)	58	$1.81x10^{-6}$	0.99	0.033	***CFAP44,*** *SPICE1*
1 (2)	58	$1.61x10^{-6}$	0.98	0.033	***CFAP44,*** *SPICE1*
1 (2)	58	$7.37x10^{-4}$	0.94	0.022	***SPICE1,*** *CFAP44*
1 (2)	58	$7.20x10^{-4}$	0.94	0.022	***SPICE1,*** *CFAP44*
1 (2)	58	$7.04x10^{-4}$	0.94	0.022	***SPICE1,*** *CFAP44*
1 (2)	58	$6.88x10^{-4}$	0.98	0.022	***SPICE1,*** *CFAP44*
1 (2)	58	$1.45x10^{-6}$	0.99	0.033	***SIDT1,*** *SPICE1*
1 (2)	58	$1.32x10^{-6}$	0.99	0.033	***SIDT1,*** *SPICE1*
1 (2)	58	$1.21x10^{-6}$	0.99	0.033	***SIDT1,*** *SPICE1*
1 (2)	58	$1.11x10^{-6}$	0.99	0.033	***SIDT1***
1 (2)	58	$1.03x10^{-6}$	0.99	0.033	***SIDT1***
1 (2)	58	$9.65x10^{-7}$	0.99	0.033	***SIDT1***
1 (3)	62	$4.09x10^{-3}$	0.01	0.017	-
1 (4)	63	$4.07x10^{-3}$	0.98	0.017	-
1 (4)	63	$4.05x10^{-3}$	0.98	0.017	-
1 (4)	63	$4.04x10^{-3}$	0.99	0.017	-
1 (5)	64	$4.02x10^{-3}$	0.99	0.017	***B4GALT4,*** *UPK1B*
1 (5)	64	$4.01x10^{-3}$	0.99	0.017	***B4GALT4,*** *UPK1B*
1 (6)	64	$9.05x10^{-7}$	0.98	0.033	***CD80,*** *TIMMDC1, LOC112447304, ADPRH*
1 (6)	64	$8.52x10^{-7}$	0.98	0.033	***ADPRH,*** *CD80, PLA1A*
1 (7)	76	$4.49x10^{-2}$	0.97	0.012	-
1 (8)	80	$3.57x10^{-3}$	0.04	0.017	***MASP1,*** *RTP1*

1 (8)	80	3.56×10^{-3}	0.96	0.017	***MASP1,*** *RTP1*
1 (8)	80	3.55×10^{-3}	0.04	0.017	***MASP1,*** *RTP1*
1 (8)	80	3.54×10^{-3}	0.04	0.017	***MASP1,*** *RTP1*
1 (9)	97	2.00×10^{-2}	0.88	0.013	*SAMD7, LRRC31*
1 (9)	97	7.47×10^{-3}	0.99	0.015	*LRRC31*
1 (9)	97	7.46×10^{-3}	0.99	0.015	*LRRC31*
1(9)	97	7.44×10^{-3}	0.99	0.015	*LRRC31*
1 (10)	127	4.57×10^{-2}	0.95	0.012	***GRK7***
1 (10)	127	1.41×10^{-2}	0.95	0.014	***GRK7***
1 (10)	127	4.56×10^{-2}	0.95	0.012	***GRK7***
1 (10)	127	4.55×10^{-2}	0.95	0.012	***GRK7***
1 (10)	127	3.21×10^{-2}	0.95	0.013	*GRK7, LOC112448292*
1 (10)	127	3.21×10^{-2}	0.95	0.013	*GRK7, LOC112448292, RNF7*
1 (10)	127	3.20×10^{-2}	0.95	0.013	*GRK7, LOC112448292, RNF7*
1 (10)	127	3.20×10^{-2}	0.95	0.013	*GRK7, LOC112448292, RNF7*
1 (10)	127	3.19×10^{-2}	0.95	0.013	*GRK7, LOC112448292, RNF7, LOC104968752*
1 (10)	127	4.55×10^{-2}	0.95	0.012	*GRK7, LOC112448292, RNF7, LOC104968752*
1 (10)	127	3.19×10^{-2}	0.95	0.013	*GRK7, LOC112448292, RNF7, LOC104968752*
1 (10)	127	4.54×10^{-2}	0.95	0.012	*GRK7, LOC112448292, RNF7, LOC104968752*
1 (10)	127	4.54×10^{-2}	0.95	0.012	*GRK7, LOC112448292, RNF7, LOC104968752*
1 (10)	127	4.53×10^{-2}	0.95	0.012	***RNF7,*** *GRK7, LOC112448292, LOC104968752*
1 (10)	127	4.53×10^{-2}	0.95	0.012	***RNF7,*** *LOC112448292, LOC104968752*
1 (10)	127	4.52×10^{-2}	0.95	0.012	*RNF7, LOC112448292, LOC104968752, LOC112448294*
1 (10)	127	4.52×10^{-2}	0.95	0.012	*RNF7, LOC112448292, LOC104968752, LOC112448294*
1 (10)	127	4.51×10^{-2}	0.95	0.012	*RNF7, LOC112448292, LOC104968752, LOC112448294*
1 (10)	127	4.51×10^{-2}	0.95	0.012	***LOC104968752,*** *RNF7, LOC112448292, LOC112448294*
1 (10)	128	1.88×10^{-2}	0.91	0.013	-
1 (10)	128	1.48×10^{-2}	0.88	0.014	-
1 (11)	128	5.30×10^{-3}	0.71	0.018	***CLSTN2***
1 (11)	128	2.63×10^{-2}	0.63	0.013	***CLSTN2***
1 (11)	128	2.63×10^{-2}	0.63	0.013	***CLSTN2***
1 (11)	128	3.14×10^{-2}	0.71	0.013	***CLSTN2***
1 (11)	128	3.14×10^{-2}	0.71	0.013	***CLSTN2***
1 (11)	128	3.34×10^{-2}	0.72	0.012	***CLSTN2***
1 (11)	128	3.14×10^{-2}	0.71	0.013	***CLSTN2***
1 (11)	128	3.33×10^{-2}	0.72	0.012	***CLSTN2***

1 (11)	128	$1.10x10^{-2}$	0.68	0.014	***CLSTN2***
1 (11)	128	$1.10x10^{-2}$	0.68	0.014	***CLSTN2***
1 (11)	128	$1.09x10^{-2}$	0.68	0.014	***CLSTN2***
1 (11)	128	$1.09x10^{-2}$	0.68	0.014	***CLSTN2***
1 (11)	128	$1.09x10^{-2}$	0.68	0.014	***CLSTN2***
1 (11)	128	$1.13x10^{-2}$	0.68	0.014	***CLSTN2***
1 (11)	128	$1.12x10^{-2}$	0.68	0.014	***CLSTN2***
1 (11)	128	$4.07x10^{-2}$	0.63	0.012	***CLSTN2***
1 (11)	128	$4.07x10^{-2}$	0.63	0.012	***CLSTN2***
1 (11)	128	$4.06x10^{-2}$	0.63	0.012	***CLSTN2***
1 (11)	128	$4.06x10^{-2}$	0.63	0.012	***CLSTN2***
1 (11)	128	$4.05x10^{-2}$	0.63	0.012	***CLSTN2***
1 (11)	128	$4.05x10^{-2}$	0.63	0.012	***CLSTN2***
1 (11)	128	$4.04x10^{-2}$	0.63	0.012	***CLSTN2***
1 (11)	128	$4.04x10^{-2}$	0.63	0.012	***CLSTN2***
1 (11)	128	$2.19x10^{-2}$	0.63	0.013	***CLSTN2***
1 (11)	129	$4.03x10^{-2}$	0.63	0.012	***CLSTN2***
1 (11)	129	$4.03x10^{-2}$	0.63	0.012	***CLSTN2***
1 (11)	129	$4.02x10^{-2}$	0.63	0.012	***CLSTN2***
1 (11)	129	$4.02x10^{-2}$	0.63	0.012	***CLSTN2***
1 (11)	129	$4.52x10^{-2}$	0.63	0.012	***CLSTN2***
1 (11)	129	$4.01x10^{-2}$	0.63	0.012	***CLSTN2***
1 (11)	129	$4.01x10^{-2}$	0.63	0.012	***CLSTN2***
1 (11)	129	$4.00x10^{-2}$	0.63	0.012	***CLSTN2***
1 (11)	129	$4.00x10^{-2}$	0.63	0.012	***CLSTN2***
1 (11)	129	$1.52x10^{-2}$	0.62	0.014	***CLSTN2***
1 (12)	141	$5.06x10^{-3}$	0.99	0.016	*TMPRSS2*
1 (13)	141	$5.09x10^{-3}$	0.96	0.016	*TMPRSS2*
1 (13)	141	$5.08x10^{-3}$	0.96	0.016	*TMPRSS2*
1 (13)	141	$5.07x10^{-3}$	0.96	0.016	*TMPRSS2*
1 (13)	141	$5.05x10^{-3}$	0.99	0.016	-
1 (14)	146	$4.55x10^{-3}$	0.97	0.018	-
2 (15)	1.4	$1.36x10^{-2}$	0.97	0.014	*AMER3*
2 (15)	1.4	$1.36x10^{-2}$	0.97	0.014	*AMER3*
2 (16)	15	$7.04x10^{-3}$	0.99	0.015	*LOC100300383*
2 (17)	91	$7.43x10^{-3}$	0.98	0.015	***CARF,*** *WDR12*
2 (17)	91	$7.41x10^{-3}$	0.98	0.015	***CARF,*** *WDR12*
2 (17)	91	$7.40x10^{-3}$	0.97	0.015	***CARF,*** *WDR12*
2 (17)	91	$7.38x10^{-3}$	0.98	0.015	***CARF,*** *WDR12, LOC783376*
2 (17)	91	$7.37x10^{-3}$	0.98	0.015	***CARF,*** *LOC783376*
2 (18)	109	$4.16x10^{-3}$	0.94	0.017	-
2 (18)	109	$4.13x10^{-3}$	0.94	0.017	-
2 (18)	109	$4.11x10^{-3}$	0.94	0.017	-
2 (18)	109	$4.09x10^{-3}$	0.99	0.017	-
2 (18)	109	$4.06x10^{-3}$	0.94	0.017	-
2 (18)	109	$4.04x10^{-3}$	0.99	0.017	-

2 (18)	109	4.02×10^{-3}	0.99	0.017	-
2 (18)	109	3.99×10^{-3}	0.99	0.017	-
2 (18)	109	3.97×10^{-3}	0.99	0.017	-
2 (19)	123	7.11×10^{-3}	0.99	0.015	-
2 (20)	135	7.09×10^{-3}	0.96	0.015	***PADI4,*** *PADI6*
2 (20)	135	7.08×10^{-3}	0.97	0.015	*SPATA21, NECAP2, CROCC*
3 (21)	20	3.28×10^{-3}	0.97	0.017	***RPRD2,*** *ECM1, TARS2*
3 (21)	20	2.12×10^{-2}	0.94	0.013	*MRPS21, CIART, C3H1orf54, APH1A, CA14*
3 (22)	56	3.99×10^{-3}	0.98	0.017	-
3 (22)	56	3.98×10^{-3}	0.98	0.017	-
3 (22)	56	3.96×10^{-3}	0.98	0.017	-
3 (22)	56	3.94×10^{-3}	0.98	0.017	-
3 (22)	56	3.93×10^{-3}	0.98	0.017	-
3 (22)	56	3.91×10^{-3}	0.98	0.017	-
3 (22)	56	3.90×10^{-3}	0.98	0.017	-
3 (22)	56	3.88×10^{-3}	0.98	0.017	-
3 (22)	56	3.87×10^{-3}	0.98	0.017	*LOC101903905*
3 (22)	56	3.85×10^{-3}	0.98	0.017	*LOC112446095*
3 (22)	56	3.84×10^{-3}	0.98	0.017	*LOC112446095*
3 (22)	56	3.82×10^{-3}	0.98	0.017	*LOC112446000*
3 (22)	56	3.81×10^{-3}	0.98	0.017	*LOC112446000, HS2ST1*
3 (22)	56	3.80×10^{-3}	0.98	0.017	*LOC112446000, HS2ST1, LOC112445906*
3 (22)	56	3.78×10^{-3}	0.98	0.017	*LOC112446000, HS2ST1, LOC112445906*
3 (22)	56	3.77×10^{-3}	0.98	0.017	*LOC112446000, HS2ST1, LOC112445906*
3 (22)	57	3.75×10^{-3}	0.98	0.017	*LOC112446001, CLCA1, CLCA2*
3 (22)	57	3.74×10^{-3}	0.98	0.017	*LOC112446001, CLCA1, CLCA2*
3 (23)	62	3.73×10^{-3}	0.98	0.017	**-**
3 (23)	62	3.71×10^{-3}	0.98	0.017	**-**
3 (24)	62	3.70×10^{-3}	0.99	0.017	***ADGRL2***
3 (24)	63	3.68×10^{-3}	0.99	0.017	*LOC112445881*
3 (24)	63	3.67×10^{-3}	0.99	0.017	***LOC112445881***
3 (25)	63	3.66×10^{-3}	0.99	0.017	*LOC112445881*
3 (25)	65	3.65×10^{-3}	0.99	0.017	***ADGRL4***
3 (25)	65	3.63×10^{-3}	0.99	0.017	***ADGRL4***
3(25)	65	3.62×10^{-3}	0.99	0.017	***ADGRL4***
3 (25)	65	3.61×10^{-3}	0.99	0.017	***ADGRL4***
3 (26)	66	3.59×10^{-3}	0.99	0.017	***PTGFR,*** *LOC104971683*
3 (26)	66	3.58×10^{-3}	0.99	0.017	***PTGFR***
3 (27)	82	2.37×10^{-2}	0.78	0.013	-
4 (28)	18	4.31×10^{-2}	0.88	0.012	-
4 (28)	18	1.62×10^{-2}	0.85	0.014	-
4 (28)	18	3.37×10^{-2}	0.85	0.012	-
4 (28)	18	3.36×10^{-2}	0.85	0.012	-
4 (28)	18	3.36×10^{-2}	0.85	0.012	-
4 (28)	18	3.35×10^{-2}	0.85	0.012	-

4 (28)	18	3.35×10^{-2}	0.85	0.012	-
4 (28)	18	3.34×10^{-2}	0.85	0.012	-
4 (28)	18	3.34×10^{-2}	0.85	0.012	-
4 (28)	18	2.35×10^{-2}	0.85	0.013	-
4 (28)	18	2.34×10^{-2}	0.85	0.013	-
4 (28)	18	2.34×10^{-2}	0.85	0.013	-
4 (28)	18	2.34×10^{-2}	0.85	0.013	-
4 (28)	18	4.71×10^{-2}	0.84	0.012	-
4 (28)	18	4.71×10^{-2}	0.84	0.012	-
4 (28)	18	3.99×10^{-2}	0.84	0.012	-
4 (28)	18	4.70×10^{-2}	0.84	0.012	-
4 (28)	18	3.99×10^{-2}	0.84	0.012	-
4 (28)	18	5.31×10^{-3}	0.82	0.016	*LOC100297616*
4 (28)	18	4.30×10^{-2}	0.88	0.012	***LOC100297616***
4 (28)	18	5.82×10^{-3}	0.81	0.016	***LOC100297616***
4 (28)	18	5.80×10^{-3}	0.81	0.016	***LOC100297616***
4 (28)	18	5.79×10^{-3}	0.82	0.016	***LOC100297616***
4 (28)	18	5.78×10^{-3}	0.82	0.016	***LOC100297616***
4 (28)	18	5.76×10^{-3}	0.82	0.016	***LOC100297616***
4 (28)	18	5.75×10^{-3}	0.81	0.016	***LOC100297616***
4 (28)	18	5.74×10^{-3}	0.81	0.016	***LOC100297616***
4 (28)	18	5.73×10^{-3}	0.81	0.016	***LOC100297616***
4 (28)	18	5.71×10^{-3}	0.81	0.016	***LOC100297616***
4 (28)	19	4.26×10^{-3}	0.83	0.018	-
4 (29)	21	8.26×10^{-7}	0.94	0.033	-
4 (29)	21	7.82×10^{-7}	0.94	0.033	-
4 (29)	21	4.47×10^{-3}	0.98	0.017	-
4 (29)	21	4.45×10^{-3}	0.98	0.017	-
4 (29)	21	4.43×10^{-3}	0.98	0.017	-
4 (29)	21	9.58×10^{-4}	0.97	0.021	*LOC112446494*
4 (29)	22	4.41×10^{-3}	0.98	0.017	***ETV1***
4 (29)	22	4.39×10^{-3}	0.98	0.017	*ETV1*
4 (30)	22	2.18×10^{-2}	0.96	0.013	-
4 (30)	22	4.37×10^{-3}	0.98	0.017	-
4 (30)	22	4.35×10^{-3}	0.98	0.017	-
4 (30)	22	4.33×10^{-3}	0.98	0.017	-
4 (30)	22	4.31×10^{-3}	0.98	0.017	-
4 (30)	22	4.29×10^{-3}	0.98	0.017	-
4 (30)	22	4.27×10^{-3}	0.98	0.017	-
4 (30)	22	4.25×10^{-3}	0.98	0.017	-
4 (30)	22	4.23×10^{-3}	0.98	0.017	-
4 (30)	22	4.22×10^{-3}	0.98	0.017	***DGKB***
4 (30)	22	4.20×10^{-3}	0.98	0.017	***DGKB***
4 (30)	22	4.18×10^{-3}	0.98	0.017	***DGKB***
4 (30)	22	4.16×10^{-3}	0.98	0.017	***DGKB***
4 (30)	22	4.14×10^{-3}	0.98	0.017	***DGKB***

4 (30)	22	4.12×10^{-3}	0.98	0.017	***DGKB***
4 (30)	22	4.11×10^{-3}	0.98	0.017	***DGKB***
4 (30)	22	4.09×10^{-3}	0.98	0.017	***DGKB***
4 (31)	22	3.98×10^{-2}	0.63	0.012	***DGKB***
4 (31)	22	5.62×10^{-3}	0.75	0.019	***DGKB***
4 (32)	30	5.08×10^{-3}	0.05	0.016	***LOC104968411,*** *LOC101907567*
4 (32)	30	5.06×10^{-3}	0.05	0.016	***LOC104968411,*** *LOC101907567*
4 (32)	30	5.05×10^{-3}	0.05	0.016	***LOC104968411***
4 (32)	30	5.04×10^{-3}	0.05	0.016	***LOC104968411***
4 (33)	48	4.75×10^{-3}	0.83	0.016	***COG5,*** *HBP1*
4 (33)	48	2.54×10^{-2}	0.73	0.013	***COG5***
4 (33)	48	4.49×10^{-2}	0.70	0.012	***COG5***
4 (33)	48	2.45×10^{-2}	0.73	0.013	***COG5***
4 (33)	48	2.44×10^{-2}	0.73	0.013	***COG5***
4 (33)	48	2.44×10^{-2}	0.73	0.013	***COG5***
4 (33)	48	2.44×10^{-2}	0.73	0.013	***COG5***
4 (33)	48	2.43×10^{-2}	0.73	0.013	***COG5***
4 (33)	48	3.32×10^{-2}	0.72	0.013	***COG5***
4 (33)	48	2.43×10^{-2}	0.73	0.013	***COG5***
4 (33)	48	4.70×10^{-2}	0.88	0.012	*BCAP29, LOC112446328, SLC26A4*
4 (33)	48	4.69×10^{-2}	0.88	0.012	*BCAP29, LOC112446328, SLC26A4*
4 (34)	49	2.99×10^{-2}	0.79	0.013	***NRCAM***
4 (35)	52	2.76×10^{-2}	0.91	0.013	-
4 (35)	52	1.20×10^{-2}	0.92	0.014	-
4 (35)	52	1.20×10^{-2}	0.92	0.014	-
4 (36)	62	4.74×10^{-2}	0.95	0.012	*BMPER*
4 (37)	111	7.02×10^{-3}	0.98	0.015	***CNTNAP2***
4 (38)	115	4.10×10^{-3}	0.98	0.017	***ACTR3B***
4 (38)	115	4.08×10^{-3}	0.97	0.017	***ACTR3B***
4 (39)	115	1.34×10^{-2}	0.93	0.014	*LOC509941*
4 (40)	117	2.22×10^{-2}	0.68	0.013	***LOC112446461***
4 (40)	117	2.21×10^{-2}	0.68	0.013	***LOC112446461***
4 (40)	117	2.21×10^{-2}	0.68	0.013	***LOC112446461***
4 (40)	117	2.21×10^{-2}	0.68	0.013	***LOC112446461***
4 (40)	117	2.20×10^{-2}	0.68	0.013	***LOC112446461***
4 (40)	117	2.20×10^{-2}	0.68	0.013	***LOC112446461***
5 (41)	11	4.62×10^{-2}	0.67	0.012	-
5 (41)	11	1.45×10^{-2}	0.67	0.014	-
5 (41)	11	4.39×10^{-2}	0.67	0.012	-
5 (42)	13	1.48×10^{-3}	0.98	0.014	-
5 (43)	13	7.63×10^{-3}	0.99	0.015	-
5 (43)	13	7.61×10^{-3}	0.99	0.015	-
5 (43)	13	7.60×10^{-3}	0.99	0.015	-
5 (43)	13	7.58×10^{-3}	0.99	0.015	-
5 (43)	13	7.57×10^{-3}	0.99	0.015	-
5 (43)	13	7.55×10^{-3}	0.99	0.015	-

5 (44)	20	1.33×10^{-2}	0.83	0.014	-
5 (44)	20	4.12×10^{-2}	0.85	0.012	-
5 (44)	20	4.70×10^{-2}	0.81	0.012	-
5 (44)	20	4.70×10^{-2}	0.81	0.012	-
5 (45)	44	7.42×10^{-4}	0.95	0.022	***CPSF6,*** *TRNAC-GCA*
5 (46)	85	3.95×10^{-3}	0.98	0.017	***SOX5***
5 (47)	111	5.56×10^{-3}	0.95	0.018	***CACNA1I,*** *LOC112441544*
5 (47)	111	3.20×10^{-6}	0.98	0.035	***ENTHD1,*** *GRAP2*
5 (48)	115	4.75×10^{-3}	0.99	0.016	*NUP50*
6 (49)	15	3.21×10^{-2}	0.96	0.013	-
6 (49)	15	3.21×10^{-2}	0.96	0.013	-
6 (50)	43	3.30×10^{-2}	0.57	0.013	-
6 (50)	43	3.47×10^{-2}	0.57	0.012	-
6 (50)	43	3.46×10^{-2}	0.57	0.012	-
6 (50)	43	3.46×10^{-2}	0.57	0.012	-
6 (50)	43	4.08×10^{-2}	0.57	0.012	-
6 (50)	43	4.08×10^{-2}	0.57	0.012	-
6 (50)	43	4.07×10^{-2}	0.57	0.012	-
6 (50)	43	3.98×10^{-2}	0.57	0.012	-
6 (50)	43	4.51×10^{-2}	0.56	0.012	-
6 (50)	43	4.51×10^{-2}	0.56	0.012	-
6 (51)	45	4.16×10^{-2}	0.99	0.012	-
6 (52)	46	4.15×10^{-2}	0.98	0.012	*TBC1D19*
6 (53)	46	3.34×10^{-2}	0.94	0.012	*LOC112447154, STIM2*
6 (54)	51	4.15×10^{-2}	0.99	0.012	-
6 (54)	51	4.14×10^{-2}	0.99	0.012	-
6 (54)	51	4.14×10^{-2}	0.99	0.012	-
6 (54)	51	4.13×10^{-2}	0.99	0.012	-
6 (54)	51	4.13×10^{-2}	0.99	0.012	-
6 (54)	51	4.12×10^{-2}	0.99	0.012	-
6 (54)	51	4.12×10^{-2}	0.99	0.012	-
6 (54)	51	4.11×10^{-2}	0.99	0.012	-
6 (54)	52	4.11×10^{-2}	0.99	0.012	-
6 (54)	52	4.10×10^{-2}	0.99	0.012	-
6 (54)	52	4.10×10^{-2}	0.99	0.012	-
6 (54)	53	4.09×10^{-2}	0.99	0.012	-
6 (54)	53	4.09×10^{-2}	0.99	0.012	-
6 (54)	53	4.08×10^{-2}	0.99	0.012	-
6 (54)	53	4.08×10^{-2}	0.99	0.012	-
6 (54)	53	4.07×10^{-2}	0.99	0.012	-
6 (54)	53	4.07×10^{-2}	0.99	0.012	-
6 (54)	53	4.06×10^{-2}	0.99	0.012	-
6 (54)	53	4.06×10^{-2}	0.98	0.012	-
6 (54)	54	4.05×10^{-2}	0.99	0.012	-
6 (55)	59	3.57×10^{-3}	0.98	0.017	***RBM47, LOC101901948***
6 (55)	59	3.56×10^{-3}	0.98	0.017	***RBM47, LOC101901948***

6 (56)	71	$2.93x10^{-3}$	0.98	0.020	
6 (56)	72	$4.05x10^{-2}$	0.98	0.012	*LOC112447194, SPINK2*
6 (56)	72	$4.04x10^{-2}$	0.98	0.012	*LOC112447194, SPINK2*
6 (56)	72	$4.04x10^{-2}$	0.98	0.012	*LOC112447194, SPINK2*
6 (56)	72	$4.03x10^{-2}$	0.98	0.012	*LOC112447194, SPINK2*
6 (56)	72	$4.03x10^{-2}$	0.98	0.012	*REST, LOC783045*
6 (57)	80	$7.36x10^{-3}$	0.97	0.015	-
6 (57)	80	$7.34x10^{-3}$	0.98	0.015	-
6 (57)	80	$7.33x10^{-3}$	0.98	0.015	-
6 (57)	80	$7.31x10^{-3}$	0.99	0.015	-
6 (57)	80	$7.30x10^{-3}$	0.99	0.015	-
6 (57)	80	$7.29x10^{-3}$	0.99	0.015	***EPHA5***
6 (57)	80	$7.27x10^{-3}$	0.99	0.015	***EPHA5***
6 (57)	81	$7.26x10^{-3}$	0.99	0.015	-
6 (58)	86	$7.06x10^{-3}$	0.04	0.015	*LOC782958*
6 (58)	86	$7.05x10^{-3}$	0.04	0.015	*LOC782958*
6 (59)	115	$4.48x10^{-3}$	0.98	0.017	*HMX1*
6 (59)	115	$4.45x10^{-3}$	0.98	0.017	*HMX1*
6 (59)	115	$4.43x10^{-3}$	0.97	0.017	*ADRA2C*
7 (60)	1.1	$4.13x10^{-2}$	0.81	0.012	***RNF130***
7 (60)	1.1	$8.80x10^{-3}$	0.75	0.015	***RNF130***
7 (60)	1.1	$7.57x10^{-3}$	0.76	0.015	***RNF130***
7 (60)	1.2	$1.76x10^{-2}$	0.75	0.014	***RNF130***
7 (61)	2.3	$7.54x10^{-3}$	0.99	0.015	***ZNF354C,*** *ZNF879, LOC112447642*
7 (61)	2.3	$7.52x10^{-3}$	0.99	0.015	***ZNF354C,*** *ZNF879, LOC112447642*
7 (62)	6.7	$3.35x10^{-2}$	0.96	0.012	***AP1M1,*** *LOC112447625*
7 (62)	6.8	$3.35x10^{-2}$	0.96	0.012	***AP1M1,*** *LOC112447625, FAM32A*
7 (62)	6.8	$3.34x10^{-2}$	0.96	0.012	***AP1M1,*** *LOC112447625, FAM32A*
7 (62)	6.8	$3.34x10^{-2}$	0.96	0.012	***AP1M1,*** *LOC112447625, FAM32A*
7 (62)	6.8	$3.33x10^{-2}$	0.96	0.012	***AP1M1,*** *LOC112447625, FAM32A*
7 (62)	6.8	$3.33x10^{-2}$	0.96	0.012	***AP1M1,*** *LOC112447625, FAM32A*
7 (62)	6.8	$3.33x10^{-2}$	0.96	0.012	***AP1M1,*** *LOC112447625, FAM32A*
7 (62)	6.8	$3.32x10^{-2}$	0.96	0.012	***AP1M1,*** *LOC112447625, FAM32A*
7 (62)	6.8	$3.32x10^{-2}$	0.96	0.012	***AP1M1,*** *LOC112447625, FAM32A*
7 (63)	14	$7.51x10^{-3}$	0.98	0.015	*PIN1, OLFM2, LOC112447606, LOC112447604*
7 (64)	17	$8.71x10^{-3}$	0.86	0.015	***LOC112447356,*** *LOC100337081, LOC518134*
7 (64)	17	$8.70x10^{-3}$	0.86	0.015	***LOC112447356,*** *LOC100337081, LOC518134*
7 (64)	17	$8.68x10^{-3}$	0.87	0.015	***LOC112447356,*** *LOC100337081, LOC518134*
7 (65)	26	$1.52x10^{-2}$	0.95	0.014	***LOC107132618, CTXN3***
7 (65)	26	$1.52x10^{-2}$	0.96	0.014	***LOC107132618,*** *CTXN3*
7 (65)	27	$3.23x10^{-2}$	0.94	0.013	***MARCHF3***
7 (66)	29	$3.70x10^{-2}$	0.88	0.012	***LOC112447389***

7 (67)	32	3.21×10^{-2}	0.97	0.013	***LOC112447394***
7 (68)	89	7.24×10^{-3}	0.98	0.015	-
7 (68)	89	7.23×10^{-3}	0.98	0.015	-
7 (68)	89	7.22×10^{-3}	0.98	0.015	-
7 (68)	89	7.20×10^{-3}	0.98	0.015	-
7 (69)	103	3.54×10^{-3}	0.96	0.017	-
8 (70)	8.2	3.53×10^{-3}	0.98	0.017	***XKR6***
8 (70)	8.2	3.52×10^{-3}	0.98	0.017	***XKR6***
8 (71)	11	4.09×10^{-3}	0.98	0.018	***LOC104969267***
8 (72)	31	3.55×10^{-3}	0.97	0.017	***MPDZ***
8 (73)	35	3.91×10^{-3}	0.88	0.012	***LOC101904827***
8(74)	63	5.94×10^{-3}	0.91	0.016	*ANKS6*
8 (74)	63	2.71×10^{-2}	0.91	0.013	*ANKS6, GALNT12*
8 (75)	73	3.46×10^{-2}	0.92	0.012	***DPYSL2,*** *LOC112447896*
8 (76)	97	5.02×10^{-3}	0.97	0.016	-
8 (77)	97	5.01×10^{-3}	0.97	0.016	-
8 (78)	97	7.19×10^{-3}	0.95	0.015	-
8 (78)	97	7.18×10^{-3}	0.95	0.015	-
8 (78)	97	7.16×10^{-3}	0.95	0.015	-
8 (79)	105	4.99×10^{-3}	0.96	0.016	*PAPPA*
8 (79)	105	4.98×10^{-3}	0.97	0.016	*PAPPA*
8 (79)	105	4.96×10^{-3}	0.96	0.016	*PAPPA*
8 (80)	105	4.95×10^{-3}	0.96	0.016	***PAPPA***
8 (80)	105	4.94×10^{-3}	0.96	0.016	***PAPPA***
8 (80)	105	4.93×10^{-3}	0.96	0.016	***PAPPA***
9 (81)	7.8	4.91×10^{-3}	0.98	0.016	***ADGRB3***
9 (81)	7.8	4.90×10^{-3}	0.98	0.016	***ADGRB3***
9 (82)	12	4.50×10^{-3}	0.97	0.018	***KCNQ5,*** *LOC112448015, LOC112448016*
9 (82)	12	4.46×10^{-3}	0.97	0.018	***KCNQ5,*** *LOC112448015, LOC112448016*
9 (83)	12	7.30×10^{-3}	0.99	0.015	***KCNQ5***
9 (83)	12	7.29×10^{-3}	0.99	0.015	***KCNQ5***
9 (83)	12	7.27×10^{-3}	0.99	0.015	***KCNQ5***
9 (83)	12	7.25×10^{-3}	0.99	0.015	***KCNQ5***
9 (83)	12	7.24×10^{-3}	0.99	0.015	***KCNQ5***
9 (83)	12	7.22×10^{-3}	0.99	0.015	***KCNQ5***
9 (83)	12	7.21×10^{-3}	0.98	0.015	*LOC104972957, KHDC3L, OOEP, DDX43*
9 (83)	12	7.19×10^{-3}	0.99	0.015	*DDX43, LOC104972961*
9 (84)	13	4.42×10^{-3}	0.99	0.018	*CD109*
9 (85)	33	7.49×10^{-3}	0.98	0.015	***ROS1***
9 (86)	48	5.08×10^{-3}	0.99	0.018	***GRIK2***
10 (87)	3.8	2.06×10^{-2}	0.77	0.013	*LOC787395*
10 (87)	3.8	1.15×10^{-2}	0.83	0.014	*LOC787395*
10 (87)	3.9	9.20×10^{-3}	0.83	0.015	*LOC787395*
10 (87)	3.9	9.18×10^{-3}	0.83	0.015	*LOC787395*
10 (87)	3.9	9.16×10^{-3}	0.83	0.015	***LOC787395***
10 (87)	3.9	4.64×10^{-2}	0.75	0.012	*TRIM36*

10 (88)	44	$1.36x10^{-2}$	0.98	0.014	*LOC783735*
10 (89)	67	$4.02x10^{-2}$	0.04	0.012	***GCH1***
10 (89)	67	$4.02x10^{-2}$	0.04	0.012	***GCH1***
10 (89)	67	$4.01x10^{-2}$	0.04	0.012	*GCH1*
10 (89)	67	$4.01x10^{-2}$	0.04	0.012	*GCH1*
10 (89)	67	$4.00x10^{-2}$	0.04	0.012	*GCH1*
10 (89)	67	$4.00x10^{-2}$	0.04	0.012	*GCH1*
10 (89)	67	$3.99x10^{-2}$	0.04	0.012	*GCH1*
10 (89)	67	$3.99x10^{-2}$	0.04	0.012	*GCH1*
10 (89)	67	$3.98x10^{-2}$	0.04	0.012	*GCH1*
10 (89)	67	$3.98x10^{-2}$	0.04	0.012	*GCH1, WDHD1*
10 (89)	67	$3.97x10^{-2}$	0.04	0.012	*WDHD1*
10 (90)	77	$4.15x10^{-2}$	0.97	0.012	*FUT8*
10 (90)	77	$4.14x10^{-2}$	0.97	0.012	***FUT8***
10 (90)	77	$4.14x10^{-2}$	0.97	0.012	***FUT8***
10 (90)	77	$4.13x10^{-2}$	0.97	0.012	***FUT8***
10 (90)	77	$4.13x10^{-2}$	0.97	0.012	***FUT8***
10 (90)	77	$4.12x10^{-2}$	0.97	0.012	***FUT8***
10 (90)	77	$4.11x10^{-2}$	0.97	0.012	***FUT8***
10 (90)	77	$4.11x10^{-2}$	0.97	0.012	***FUT8***
10 (90)	77	$4.10x10^{-2}$	0.97	0.012	***FUT8***
10 (90)	77	$4.10x10^{-2}$	0.97	0.012	***FUT8***
10 (91)	80	$3.97x10^{-2}$	0.99	0.012	***RAD51B***
10 (92)	86	$3.96x10^{-2}$	0.02	0.012	***YLPM1***
10 (93)	99	$4.15x10^{-3}$	0.05	0.016	-
10 (93)	99	$4.13x10^{-3}$	0.05	0.016	-
10 (93)	99	$4.12x10^{-3}$	0.05	0.016	-
10 (93)	99	$4.11x10^{-3}$	0.05	0.016	-
10 (93)	99	$4.10x10^{-3}$	0.05	0.016	**-**
10 (94)	101	$3.96x10^{-2}$	0.98	0.012	***EFCAB11,*** *LOC101907299*
11 (95)	63	$4.56x10^{-2}$	0.83	0.012	***SERTAD2***
11 (95)	63	$4.55x10^{-2}$	0.83	0.012	***SERTAD2***
11 (96)	100	$7.48x10^{-3}$	0.96	0.015	*PTGES*
11 (97)	106	$4.89x10^{-3}$	0.02	0.016	*DPH7, ZMYND19, ARRDC1, EHMT1*
11 (97)	106	$4.88x10^{-3}$	0.02	0.016	***EHMT1***
11 (97)	106	$4.87x10^{-3}$	0.02	0.016	***EHMT1***
12 (98)	18	$1.08x10^{-2}$	0.94	0.014	***ITM2B***
12 (99)	21	$7.01x10^{-3}$	0.99	0.015	***WDFY2***
12 (100)	23	$6.99x10^{-3}$	0.98	0.015	***FREM2***
12 (100)	23	$6.98x10^{-3}$	0.98	0.015	***FREM2***
12 (100)	23	$2.09x10^{-3}$	0.85	0.020	*FREM2*
12 (100)	23	$6.96x10^{-3}$	0.98	0.015	*LOC112449153*

12 (100)	23	6.95x10^{-3}	0.98	0.015	*LOC112449153*
12 (101)	25	4.07x10^{-3}	0.98	0.017	*DCLK1*
12 (101)	25	4.06x10^{-3}	0.98	0.017	*DCLK1*
12 (101)	25	4.04x10^{-3}	0.98	0.017	*DCLK1*
12 (101)	25	4.02x10^{-3}	0.99	0.017	*NBEA*
12 (102)	27	7.40x10^{-3}	0.98	0.015	-
12 (102)	27	7.38x10^{-3}	0.98	0.015	*STARD13*
12 (102)	27	7.36x10^{-3}	0.98	0.015	***STARD13, LOC107132979***
12 (103)	30	3.97x10^{-2}	0.98	0.012	*UBL3, LOC112449055*
12 (103)	31	3.96x10^{-2}	0.98	0.012	***MTUS2***
12 (104)	53	3.48x10^{-2}	0.96	0.012	-
12 (104)	53	3.48x10^{-2}	0.96	0.012	-
12 (104)	53	3.47x10^{-2}	0.96	0.012	-
12 (104)	53	3.47x10^{-2}	0.96	0.012	-
12 (104)	53	3.46x10^{-2}	0.96	0.012	-
12 (105)	56	3.99x10^{-2}	0.93	0.012	-
12 (106)	68	1.40x10^{-2}	0.63	0.014	***GPC6***
12 (107)	79	4.00x10^{-3}	0.99	0.017	-
12 (107)	79	3.99x10^{-3}	0.99	0.017	-
12 (107)	79	3.97x10^{-3}	0.99	0.017	-
12 (107)	79	3.95x10^{-3}	0.99	0.017	-
12 (107)	79	3.94x10^{-3}	0.99	0.017	-
12 (107)	79	3.92x10^{-3}	0.99	0.017	*LOC112449139*

12 (107)	79	3.91x10^{-3}	0.99	0.017	*LOC112449139*
12 (108)	86	7.14x10^{-3}	0.97	0.015	***RASA3,*** *LOC104973683, C12H13orf46, LOC112449104*
13 (109)	3.2	3.54x10^{-3}	0.96	0.017	***ANKEF1***
13 (110)	5.5	4.59x10^{-3}	0.98	0.018	-
13 (111)	41	4.38x10^{-3}	0.94	0.018	-
13 (111)	41	4.34x10^{-3}	0.94	0.018	-
13 (111)	41	4.30x10^{-3}	0.94	0.018	-
13 (111)	41	4.26x10^{-3}	0.94	0.018	-
13 (111)	41	4.22x10^{-3}	0.94	0.018	-
13 (111)	41	4.18x10^{-3}	0.94	0.018	-
13 (111)	41	4.14x10^{-3}	0.94	0.018	-
13 (111)	41	4.11x10^{-3}	0.06	0.018	-
13 (111)	41	4.07x10^{-3}	0.94	0.018	-
13 (112)	41	4.03x10^{-3}	0.06	0.018	***LOC617402,*** *CSTL1, CST11, MGC133636, CST8, LOC531692*
13 (113)	64	4.05x10^{-3}	0.02	0.016	***PIGU,*** *LOC786401, LOC100295994, TP53INP2*
13 (114)	75	4.85x10^{-3}	0.97	0.016	***CDH22***
13 (114)	75	5.83x10^{-3}	0.95	0.018	*LOC100847115, LOC101904842*
13 (114)	75	5.74x10^{-3}	0.95	0.018	*LOC100847115, LOC101904842, OCSTAMP*
13 (114)	75	5.66x10^{-3}	0.95	0.018	*LOC100847115, LOC101904842, OCSTAMP*
13 (114)	75	5.57x10^{-3}	0.95	0.018	*SLC2A10, LOC104973899*
13 (114)	75	5.49x10^{-3}	0.95	0.018	*SLC2A10, LOC104973899*
13 (114)	75	5.42x10^{-3}	0.94	0.018	***EYA2,*** *LOC112449430*
13 (114)	75	5.34x10^{-3}	0.94	0.018	***EYA2,*** *LOC112449430*

13 (114)	75	$4.84x10^{-3}$	0.97	0.016	***EYA2***
13 (114)	75	$4.83x10^{-3}$	0.97	0.016	***EYA2***
13 (114)	75	$4.82x10^{-3}$	0.98	0.016	***EYA2***
13 (114)	75	$4.81x10^{-3}$	0.98	0.016	***EYA2***
13 (114)	75	$4.80x10^{-3}$	0.98	0.016	***EYA2***
13 (114)	75	$4.78x10^{-3}$	0.98	0.016	***EYA2***
13 (114)	75	$4.77x10^{-3}$	0.98	0.016	***EYA2***
13 (114)	75	$4.76x10^{-3}$	0.98	0.016	***EYA2***
13 (114)	75	$4.75x10^{-3}$	0.98	0.016	***EYA2***
13 (114)	75	$4.74x10^{-3}$	0.97	0.016	***EYA2***
13 (115)	75	$1.57x10^{-2}$	0.91	0.014	***EYA2,*** *LOC112449339, ZMYND8*
13 (115)	75	$7.82x10^{-3}$	0.70	0.015	*EYA2, LOC112449339, ZMYND8, LOC101905203*
13 (115)	75	$7.80x10^{-3}$	0.70	0.015	*EYA2, LOC112449339, ZMYND8, LOC101905203*
13 (115)	75	$1.57x10^{-2}$	0.91	0.014	***LOC112449339,*** *EYA2, ZMYND8, LOC101905203*
13 (115)	75	$2.34x10^{-2}$	0.79	0.013	*EYA2, LOC112449339, ZMYND8, LOC101905203*
13 (115)	75	$2.34x10^{-2}$	0.79	0.013	*EYA2, LOC112449339, ZMYND8, LOC101905203*
13 (115)	75	$3.90x10^{-2}$	0.79	0.012	***ZMYND8,*** *LOC101905203, LOC112449409, LOC104973934*
13 (115)	75	$8.05x10^{-3}$	0.68	0.015	***ZMYND8,*** *LOC112449409, LOC104973934*
13 (115)	75	$1.83x10^{-2}$	0.72	0.013	***ZMYND8***
13 (116)	77	$1.33x10^{-2}$	0.92	0.014	*ZNFX1, LOC100847759, LOC112449404, LOC112449405, LOC112449403, TRNAG-UCC*
13 (116)	77	$3.51x10^{-3}$	0.95	0.017	***SLC9A8***
13 (116)	77	$3.50x10^{-3}$	0.95	0.017	***SLC9A8***
13 (116)	77	$3.49x10^{-3}$	0.95	0.017	***SLC9A8,*** *LOC112449243, SPATA2*

13 (117)	77	$1.18x10^{-2}$	0.92	0.014	-
14 (118)	1.2	$4.96x10^{-3}$	0.96	0.016	*ZC3H3, MAFA, RHPN1*
14 (118)	1.3	$4.95x10^{-3}$	0.04	0.016	*GPIHBP1, LY6H, LY6L*
14 (119)	10	$3.51x10^{-3}$	0.99	0.017	***ASAP1***
14 (119)	10	$3.49x10^{-3}$	0.99	0.017	***ASAP1***
14 (119)	10	$3.48x10^{-3}$	0.99	0.017	***ASAP1***
14 (119)	10	$3.47x10^{-3}$	0.99	0.017	***ASAP1***
14 (120)	76	$6.94x10^{-3}$	0.96	0.015	*PSKH2*
14 (120)	76	$6.92x10^{-3}$	0.97	0.015	-
14 (120)	76	$6.91x10^{-3}$	0.97	0.015	-
14 (120)	76	$6.89x10^{-3}$	0.97	0.015	-
14 (121)	76	$6.88x10^{-3}$	0.99	0.015	*LOC104974137*
14 (121)	76	$6.87x10^{-3}$	0.99	0.015	*LOC104974137*
14 (121)	76	$6.85x10^{-3}$	0.99	0.015	*LOC104974137*
14 (122)	81	$4.62x10^{-3}$	0.96	0.017	***COL14A1***
14 (122)	81	$4.60x10^{-3}$	0.96	0.017	***COL14A1***
14 (122)	81	$4.58x10^{-3}$	0.96	0.017	***COL14A1***
14 (122)	81	$4.56x10^{-3}$	0.96	0.017	***COL14A1***
15 (123)	15	$4.64x10^{-3}$	0.98	0.017	*LOC112441704*
15 (124)	25	$1.20x10^{-3}$	0.93	0.021	-
15 (125)	32	$3.70x10^{-3}$	0.94	0.019	***LOC107131357***
15 (125)	32	$3.63x10^{-3}$	0.94	0.019	***LOC107131357***
15 (125)	32	$3.57x10^{-3}$	0.94	0.019	***LOC107131357***

15 (125)	32	$1.54x10^{-3}$	0.93	0.014	***LOC107131357***
15 (125)	32	$3.50x10^{-3}$	0.94	0.019	***LOC107131357***
15 (126)	38	$4.46x10^{-2}$	0.92	0.012	***SPON1***
15 (126)	38	$4.45x10^{-2}$	0.92	0.012	***SPON1***
15 (127)	47	$3.93x10^{-3}$	0.97	0.017	***LOC101902757, LOC788136,*** *TRIM5, LOC516599*
15 (127)	47	$3.91x10^{-3}$	0.97	0.017	***LOC101902757, LOC516599,*** *TRIM5, LOC788136, TRIM34*
15 (127)	48	$3.88x10^{-3}$	0.99	0.017	*LOC512399, LOC515619, LOC532436, OR52H1, MGC137098*
15 (128)	53	$8.92x10^{-3}$	0.97	0.015	***UCP2,*** *PAAF1, DNAJB13, LOC112441550, UCP3*
16 (129)	2.1	$3.53x10^{-3}$	0.97	0.017	*LOC101907913, PPP1R15B, LOC112441809, PIK3C2B*
16 (129)	2.1	$3.52x10^{-3}$	0.97	0.017	*LOC101907913, PPP1R15B, LOC112441809, PIK3C2B*
16 (129)	2.1	$3.51x10^{-3}$	0.97	0.017	***PIK3C2B,*** *LOC112441809*
16 (129)	2.1	$3.50x10^{-3}$	0.96	0.017	***PIK3C2B,*** *LOC112441809*
16 (129)	2.1	$3.49x10^{-3}$	0.96	0.017	***PIK3C2B,*** *LOC112441809*
16 (129)	2.2	$3.48x10^{-3}$	0.97	0.017	***PIK3C2B,*** *LOC112441896, TRNAK-UUU, MDM4*
16 (129)	2.3	$3.47x10^{-3}$	0.97	0.017	*LRRN2*
16 (129)	2.3	$3.46x10^{-3}$	0.97	0.017	*LRRN2*
16 (130)	15	$7.61x10^{-3}$	0.03	0.015	***BRINP3***
16 (131)	29	$5.00x10^{-3}$	0.95	0.016	***STUM,*** *LOC100335838, ITPKB*
16 (132)	37	$4.14x10^{-3}$	0.89	0.012	-
16 (132)	37	$4.14x10^{-2}$	0.89	0.012	-
16 (132)	37	$4.13x10^{-2}$	0.89	0.012	-
16 (132)	37	$4.13x10^{-2}$	0.89	0.012	-
16 (132)	37	$4.12x10^{-2}$	0.89	0.012	-

16 (132)	37	$4.12x10^{-2}$	0.89	0.012	-
16 (132)	37	$4.11x10^{-2}$	0.89	0.012	-
16 (132)	38	$4.10x10^{-2}$	0.89	0.012	-
16 (132)	38	$4.10x10^{-2}$	0.89	0.012	*GORAB*
16 (132)	38	$4.09x10^{-2}$	0.89	0.012	*GORAB*
16 (132)	38	$4.09x10^{-2}$	0.89	0.012	*GORAB*
16 (132)	38	$4.08x10^{-2}$	0.89	0.012	***PRRX1,*** *LOC101901943*
16 (132)	38	$4.08x10^{-2}$	0.88	0.012	***PRRX1***
16 (132)	38	$4.07x10^{-2}$	0.89	0.012	***PRRX1***
16 (132)	38	$4.07x10^{-2}$	0.89	0.012	***PRRX1***
16 (132)	38	$4.06x10^{-2}$	0.89	0.012	-
16 (132)	38	$4.06x10^{-2}$	0.89	0.012	-
16 (132)	38	$4.05x10^{-2}$	0.89	0.012	-
16 (132)	38	$4.05x10^{-2}$	0.89	0.012	-
16 (132)	38	$4.04x10^{-2}$	0.90	0.012	-
16 (132)	38	$4.03x10^{-2}$	0.89	0.012	***MROH9***
16 (132)	38	$4.03x10^{-2}$	0.89	0.012	***MROH9***
16 (132)	38	$4.02x10^{-2}$	0.89	0.012	***FMO3,*** *TRNAE-UUC, LOC517828*
16 (133)	40	$3.96x10^{-2}$	0.91	0.012	***LOC112441831,*** *LOC784385, TRNAC-ACA*
16 (134)	56	$5.12x10^{-3}$	0.05	0.016	***TNR***
16 (134)	56	$5.11x10^{-3}$	0.05	0.016	***TNR***
16 (134)	56	$5.09x10^{-3}$	0.05	0.016	***TNR***
16 (135)	57	$3.46x10^{-6}$	0.05	0.034	***LOC101902053,*** *COP1*

16 (135)	57	$5.08x10^{-3}$	0.05	0.016	*LOC100848409*
16 (136)	69	$5.16x10^{-3}$	0.99	0.016	***SMYD2,*** *PTPN14*
16 (136)	69	$5.15x10^{-3}$	0.99	0.016	***SMYD2***
16 (136)	69	$5.13x10^{-3}$	0.99	0.016	***SMYD2***
16 (136)	69	$5.12x10^{-3}$	0.99	0.016	***SMYD2***
16 (137)	73	$5.99x10^{-3}$	0.99	0.018	*HHAT*
16 (137)	73	$5.91x10^{-3}$	0.99	0.018	-
16 (138)	73	$5.83x10^{-3}$	0.98	0.018	*HSD11B1, G0S2, LOC112441869, LAMB3*
16 (138)	73	$5.76x10^{-3}$	0.99	0.018	*HSD11B1, G0S2, LOC112441869, LAMB3*
16 (139)	73	$5.69x10^{-3}$	0.07	0.018	*CAMK1G*
16 (139)	73	$5.61x10^{-3}$	0.07	0.018	*CAMK1G*
16 (139)	73	$5.54x10^{-3}$	0.07	0.018	-
16 (139)	73	$5.47x10^{-3}$	0.07	0.018	-
16 (139)	73	$5.41x10^{-3}$	0.07	0.018	-
16 (139)	73	$5.34x10^{-3}$	0.07	0.018	-
16 (139)	73	$5.28x10^{-3}$	0.07	0.018	-
16 (139)	73	$5.22x10^{-3}$	0.07	0.018	-
16 (139)	73	$5.16x10^{-3}$	0.07	0.018	-
16 (139)	73	$5.10x10^{-3}$	0.93	0.018	-
16 (139)	73	$5.04x10^{-3}$	0.07	0.018	*LOC104974529, MIR205, LOC104974530*
16 (140)	78	$3.76x10^{-3}$	0.87	0.019	-
16 (140)	78	$3.68x10^{-3}$	0.87	0.019	-
16 (140)	78	$3.61x10^{-3}$	0.87	0.019	-

16 (140)	78	2.71×10^{-3}	0.80	0.013	-
16 (140)	79	1.22×10^{-3}	0.88	0.014	-
16 (140)	79	3.45×10^{-2}	0.89	0.012	*ZNF281*
16 (140)	79	2.73×10^{-2}	0.89	0.013	*CAMSAP2*
16 (140)	79	3.35×10^{-2}	0.88	0.012	***LOC112441775,*** *LOC112441773, LOC100337507, CYB5R1, ADIPOR1, KLHL12*
16 (141)	79	2.01×10^{-2}	0.86	0.013	***KLHL12,*** *LOC112441775, LOC100337507, RABIF, ADIPOR1, CYB5R1*
16 (141)	79	3.34×10^{-2}	0.88	0.012	*LOC100337507, ADIPOR1, KLHL12, RABIF*
16 (141)	79	2.01×10^{-2}	0.86	0.013	***KDM5B***
16 (141)	79	2.01×10^{-2}	0.86	0.013	***KDM5B***
16 (141)	79	2.01×10^{-2}	0.86	0.013	***KDM5B***
16 (141)	80	2.00×10^{-2}	0.86	0.013	***KDM5B***
16 (141)	80	2.00×10^{-2}	0.86	0.013	*KDM5B, SYT2*
17 (142)	14	6.88×10^{-3}	0.91	0.015	-
17 (142)	14	4.01×10^{-2}	0.89	0.012	-
17 (143)	14	3.36×10^{-3}	0.90	0.017	-
17 (143)	14	3.35×10^{-3}	0.90	0.017	-
17 (143)	14	3.34×10^{-3}	0.90	0.017	-
17 (144)	15	7.21×10^{-3}	0.91	0.015	***LOC112442091***
17 (145)	35	4.73×10^{-3}	0.98	0.016	***LOC112442028, IL21***
17 (146)	61	5.36×10^{-3}	0.97	0.018	***TPCN1,*** *IQCD*
17 (147)	64	2.38×10^{-2}	0.94	0.013	-
18 (148)	5.1	9.00×10^{-7}	0.98	0.033	***VAT1L***
18 (148)	5.2	5.52×10^{-3}	0.99	0.017	*CLEC3A, WWOX*

18 (148)	5.3	$4.30x10^{-3}$	0.05	0.017	***WWOX***
18 (148)	5.3	$4.28x10^{-3}$	0.05	0.017	***WWOX***
18 (148)	5.3	$4.26x10^{-3}$	0.05	0.017	***WWOX***
18 (149)	5.4	$5.48x10^{-3}$	0.97	0.017	***WWOX***
18 (149)	5.4	$5.44x10^{-3}$	0.97	0.017	***WWOX***
18 (149)	5.4	$5.40x10^{-3}$	0.97	0.017	***WWOX***
18 (149)	5.5	$5.35x10^{-3}$	0.97	0.017	***WWOX***
18 (149)	5.5	$5.31x10^{-3}$	0.97	0.017	***WWOX***
18 (149)	5.5	$5.27x10^{-3}$	0.97	0.017	***WWOX***
18 (149)	5.5	$8.57x10^{-7}$	0.97	0.033	***WWOX***
18 (149)	5.5	$8.18x10^{-7}$	0.97	0.033	***WWOX***
18 (149)	5.5	$7.82x10^{-7}$	0.97	0.033	***WWOX***
18 (149)	5.5	$7.50x10^{-7}$	0.96	0.033	***WWOX***
18 (149)	5.5	$7.20x10^{-7}$	0.96	0.033	***WWOX***
18 (149)	5.5	$6.92x10^{-7}$	0.96	0.033	***WWOX***
18 (149)	5.5	$6.67x10^{-7}$	0.96	0.033	***WWOX***
18 (150)	5.5	$6.43x10^{-7}$	0.97	0.033	***WWOX***
18 (150)	5.5	$6.21x10^{-7}$	0.97	0.033	***WWOX***
18 (151)	5.8	$3.34x10^{-2}$	0.97	0.012	***WWOX***
18 (152)	6.4	$6.00x10^{-7}$	0.98	0.033	***MAF***
18 (152)	6.4	$5.81x10^{-7}$	0.98	0.033	***MAF***
18 (153)	6.5	$5.24x10^{-3}$	0.99	0.017	***LOC101902700,*** *LOC112442252*
18 (153)	6.5	$5.20x10^{-3}$	0.99	0.017	***LOC101902700,*** *LOC112442252*

18 (153)	6.5	8.57×10^{-3}	0.90	0.015	***LOC101902700,*** *LOC112442252*
18 (153)	6.5	5.16×10^{-3}	0.99	0.017	***LOC101902700,*** *LOC112442252*
18 (153)	6.5	5.62×10^{-7}	0.98	0.033	***LOC101902700,*** *LOC112442252*
18 (153)	6.5	5.12×10^{-3}	0.99	0.017	***LOC101902700***
18 (154)	7.0	9.58×10^{-6}	0.91	0.029	-
18 (154)	7.0	2.74×10^{-4}	0.97	0.024	-
18 (155)	7.1	5.45×10^{-7}	0.96	0.033	-
18 (156)	7.4	5.09×10^{-3}	0.98	0.017	***CDYL2***
18 (156)	7.4	4.05×10^{-2}	0.86	0.012	***CDYL2***
18 (156)	7.5	5.05×10^{-3}	0.98	0.017	*CDYL2*
18 (156)	7.6	5.01×10^{-3}	0.97	0.017	-
18 (156)	7.6	4.98×10^{-3}	0.98	0.017	-
18 (157)	7.6	1.10×10^{-2}	0.91	0.014	*LOC101902820, CMC2*
18 (157)	7.6	1.10×10^{-2}	0.91	0.014	*LOC101902820, CMC2*
18 (157)	7.6	1.09×10^{-2}	0.91	0.014	*LOC101902820, CMC2*
18 (157)	7.6	1.09×10^{-2}	0.91	0.014	***LOC101902820,*** *CMC2, CENPN*
18 (157)	7.6	1.09×10^{-2}	0.91	0.014	***LOC101902820,*** *CMC2, CENPN*
18 (157)	7.6	1.09×10^{-2}	0.91	0.014	***LOC101902820,*** *CMC2, CENPN*
18 (157)	7.6	1.09×10^{-2}	0.91	0.014	***LOC101902820,*** *CMC2, CENPN*
18 (157)	7.6	1.08×10^{-2}	0.91	0.014	***LOC101902820,*** *CMC2, CENPN*
18 (157)	7.9	1.08×10^{-2}	0.91	0.014	***GAN***
18 (158)	8.2	2.76×10^{-2}	0.91	0.013	***CMIP***
18 (158)	8.2	2.76×10^{-2}	0.91	0.013	***CMIP***

18 (158)	8.2	$2.75x10^{-2}$	0.91	0.013	***CMIP***
18 (158)	8.2	$2.75x10^{-2}$	0.91	0.013	***CMIP***
18 (158)	9.3	$5.86x10^{-3}$	0.76	0.016	***CDH13***
18 (158)	9.7	$1.08x10^{-2}$	0.93	0.014	***CDH13***
18 (158)	9.8	$1.08x10^{-2}$	0.93	0.014	***CDH13***
18 (159)	24	$5.65x10^{-3}$	0.94	0.018	-
18 (159)	24	$5.60x10^{-3}$	0.94	0.018	***CES5A***
18 (159)	24	$5.55x10^{-3}$	0.94	0.018	***CES5A***
18 (159)	24	$5.51x10^{-3}$	0.94	0.018	***CES5A***
18 (159)	24	$4.64x10^{-3}$	0.89	0.012	***CES5A***
18 (159)	24	$5.46x10^{-3}$	0.94	0.018	***CES5A***
18 (159)	24	$5.42x10^{-3}$	0.94	0.018	***CES5A***
18 (159)	24	$5.38x10^{-3}$	0.94	0.018	***CES5A***
18 (159)	24	$5.33x10^{-3}$	0.94	0.018	***CES5A***
18 (160)	41	$3.46x10^{-3}$	0.99	0.017	-
18 (160)	41	$3.45x10^{-3}$	0.99	0.017	-
18 (161)	44	$3.43x10^{-3}$	0.98	0.017	-
18 (161)	44	$3.42x10^{-3}$	0.98	0.017	-
18 (161)	44	$3.41x10^{-3}$	0.98	0.017	-
18 (161)	44	$3.40x10^{-3}$	0.98	0.017	-
18 (161)	44	$3.39x10^{-3}$	0.98	0.017	-
18 (161)	44	$3.38x10^{-3}$	0.98	0.017	-
18 (161)	44	$3.37x10^{-3}$	0.98	0.017	-

18 (161)	44	$3.35x10^{-3}$	0.98	0.017	-
18 (161)	44	$3.34x10^{-3}$	0.98	0.017	-
18 (161)	44	$3.33x10^{-3}$	0.99	0.017	-
18 (161)	44	$3.32x10^{-3}$	0.99	0.017	*LSM14A*
18 (162)	63	$5.11x10^{-3}$	0.99	0.016	***CNOT3,*** *MBOAT7, TMC4, LENG1, LOC101905303, PRPF31, TFPT*
18 (163)	63	$5.09x10^{-3}$	0.99	0.016	***ZNF667,*** *EDDM13, LOC101905919, ZNF583*
20 (164)	9.5	$3.48x10^{-2}$	0.89	0.012	*MAP1B*
20 (165)	57	$4.94x10^{-3}$	0.96	0.017	***FBXL7***
20 (165)	57	$4.91x10^{-3}$	0.96	0.017	***FBXL7***
20 (165)	57	$4.87x10^{-3}$	0.96	0.017	***FBXL7***
20 (166)	57	$4.84x10^{-3}$	0.96	0.017	***FBXL7***
20 (167)	58	$4.81x10^{-3}$	0.95	0.017	***ANKH***
20 (167)	58	$4.78x10^{-3}$	0.95	0.017	***ANKH***
20 (168)	58	$4.74x10^{-3}$	0.94	0.017	***OTULIN***
20 (168)	58	$4.71x10^{-3}$	0.94	0.017	***OTULINL***
20 (168)	58	$4.68x10^{-3}$	0.94	0.017	***OTULINL***
20 (168)	58	$4.65x10^{-3}$	0.94	0.017	*OTULINL*
20 (168)	58	$4.62x10^{-3}$	0.94	0.017	*OTULINL*
20 (169)	59	$4.59x10^{-3}$	0.95	0.017	*DNAH5*
20 (169)	59	$4.56x10^{-3}$	0.95	0.017	-
20 (169)	59	$4.53x10^{-3}$	0.95	0.017	-
20 (169)	59	$4.50x10^{-3}$	0.96	0.017	-
20 (169)	60	$4.47x10^{-3}$	0.96	0.017	-

20 (169)	60	4.45×10^{-3}	0.96	0.017	-
20 (170)	61	4.42×10^{-3}	0.95	0.017	***CTNND2***
20 (170)	61	4.39×10^{-3}	0.95	0.017	***CTNND2***
20 (170)	61	4.36×10^{-3}	0.95	0.017	***CTNND2***
20 (170)	61	4.34×10^{-3}	0.95	0.017	***CTNND2***
20 (170)	61	4.31×10^{-3}	0.96	0.017	***CTNND2***
20 (170)	61	4.28×10^{-3}	0.95	0.017	***CTNND2,*** *TRNAC-GCA*
20 (170)	61	4.26×10^{-3}	0.95	0.017	***CTNND2,*** *TRNAC-GCA*
20 (170)	61	4.23×10^{-3}	0.95	0.017	***CTNND2***
20 (171)	68	4.21×10^{-3}	0.97	0.017	-
20 (171)	68	4.18×10^{-3}	0.97	0.017	-
21 (172)	21	4.41×10^{-3}	0.97	0.017	***BLM,*** *LOC107131599*
21 (173)	41	1.36×10^{-2}	0.99	0.014	***LOC101908185, HEATR5A***
21 (173)	41	1.36×10^{-2}	0.99	0.014	***LOC101908185, HEATR5A***
21 (173)	41	1.36×10^{-2}	0.99	0.014	***HEATR5A,*** *LOC101908185, LOC112443378*
21 (173)	41	1.35×10^{-2}	0.99	0.014	***HEATR5A,*** *LOC101908185, LOC112443378*
21 (173)	41	1.35×10^{-2}	0.99	0.014	***HEATR5A***, *LOC112443378*
21 (173)	41	1.35×10^{-2}	0.99	0.014	***HEATR5A***, *LOC112443378*
21 (173)	41	1.35×10^{-2}	0.99	0.014	***HEATR5A***, *LOC112443378*
21 (173)	42	1.34×10^{-2}	0.98	0.014	***NUBPL,*** *LOC107131620, LOC112443346*
21 (173)	42	1.34×10^{-2}	0.99	0.014	***NUBPL,*** *LOC107131620, LOC112443346*
21 (173)	42	1.34×10^{-2}	0.98	0.014	***NUBPL,*** *LOC107131620, LOC112443346*
21 (173)	42	1.34×10^{-2}	0.98	0.014	***NUBPL,*** *LOC107131620, LOC112443346*

21 (173)	42	$1.34x10^{-2}$	0.99	0.014	***NUBPL,*** *LOC107131620, LOC112443346*
21 (173)	42	$1.33x10^{-2}$	0.99	0.014	***AKAP6***
21 (174)	58	$7.12x10^{-3}$	0.98	0.015	*DDX24, LOC514011, ISG12(B), IFI27*
21 (175)	63	$3.45x10^{-3}$	0.96	0.017	-
21 (175)	63	$3.44x10^{-3}$	0.95	0.017	-
21 (175)	63	$3.43x10^{-3}$	0.96	0.017	-
21 (175)	63	$3.42x10^{-3}$	0.95	0.017	-
21 (175)	63	$3.41x10^{-3}$	0.95	0.017	-
21 (175)	64	$3.40x10^{-3}$	0.95	0.017	*BCL11B*
21 (176)	64	$3.39x10^{-3}$	0.99	0.017	***BCL11B***
21 (176)	64	$3.38x10^{-3}$	0.97	0.017	***CCDC85C,*** *CCNK*
21 (176)	64	$3.37x10^{-3}$	0.97	0.017	***CCDC85C,*** *CCNK*
21 (176)	64	$3.36x10^{-3}$	0.99	0.017	***CCDC85C***
21 (176)	64	$3.35x10^{-3}$	0.99	0.017	***CCDC85C***
21 (176)	64	$3.34x10^{-3}$	0.99	0.017	***CCDC85C***
21 (176)	64	$3.33x10^{-3}$	0.98	0.017	*CCDC85C, HHIPL1*
21 (177)	68	$4.48x10^{-3}$	0.93	0.017	*MIR2284K, ATP5MPL, TDRD9, RD3L*
21 (177)	68	$4.46x10^{-3}$	0.93	0.017	***ATP5MPL,*** *MIR2284K, TDRD9, RD3L*
22 (178)	1	$9.42x10^{-3}$	0.92	0.015	***EGFR,*** *LOC112443476*
22 (179)	3.5	$4.05x10^{-3}$	0.02	0.016	-
22 (180)	5.5	$4.94x10^{-3}$	0.94	0.016	-
22 (180)	5.5	$4.93x10^{-3}$	0.95	0.016	-
22 (180)	5.5	$4.92x10^{-3}$	0.94	0.016	*LOC112443493*

22 (180)	5.5	4.91×10^{-3}	0.95	0.016	*LOC112443493*
22 (180)	5.5	4.90×10^{-3}	0.95	0.016	*LOC112443493*
22 (180)	5.5	4.88×10^{-3}	0.95	0.016	*LOC112443493*
22 (181)	30	4.87×10^{-3}	0.06	0.016	*LOC104976682*
22 (182)	31	4.86×10^{-3}	0.07	0.016	***LOC104976682***
23 (183)	43	4.53×10^{-3}	0.94	0.017	***PHACTR1***
23 (184)	44	4.51×10^{-3}	0.96	0.017	***ADTRP,*** *TMEM170B*
23 (185)	45	4.49×10^{-3}	0.96	0.017	*SMIM13, LOC104969833*
24 (186)	1.8	4.11×10^{-3}	0.97	0.018	-
24 (187)	43	4.09×10^{-3}	0.97	0.016	***CEP192***
24 (188)	46	5.71×10^{-3}	0.92	0.018	***KATNAL2, LOC104975783,*** *LOC112444168, LOC112444235*
24 (188)	46	5.62×10^{-3}	0.92	0.018	***KATNAL2,*** *LOC104975783, LOC112444168, LOC112444235*
24 (189)	50	7.12×10^{-3}	0.98	0.015	*SMAD4, LOC112444219*
24 (189)	50	7.11×10^{-3}	0.98	0.015	*SMAD4, LOC112444219*
24 (189)	50	7.10×10^{-3}	0.98	0.015	*SMAD4*
24 (189)	50	5.90×10^{-3}	0.89	0.016	-
24 (190)	51	4.08×10^{-3}	0.95	0.016	-
24 (190)	51	4.07×10^{-3}	0.95	0.016	-
24 (190)	51	4.06×10^{-3}	0.95	0.016	-
24 (190)	51	5.61×10^{-3}	0.97	0.018	-
24 (191)	54	3.96×10^{-3}	0.98	0.012	***TCF4***
25 (192)	5.1	1.33×10^{-3}	0.05	0.014	***RBFOX1***
25 (192)	5.8	1.33×10^{-3}	0.96	0.014	***RBFOX1***

25 (192)	5.8	1.33×10^{-3}	0.96	0.014	***RBFOX1***
25 (192)	6.2	1.32×10^{-3}	0.96	0.014	***RBFOX1,*** *LOC112444294*
25 (193)	25	7.15×10^{-3}	0.99	0.015	***ATP2A1,*** *CD19, RABEP2, LOC101908406, SH2B1, LOC107131825*
25 (194)	36	3.86×10^{-3}	0.99	0.017	*CYP3A24, LOC101904538, ZSCAN25, TMEM225B*
25 (194)	36	3.84×10^{-3}	0.99	0.017	***LOC101904538,*** *CYP3A24, ZSCAN25, TMEM225B*
25 (194)	36	3.82×10^{-3}	0.98	0.017	*LOC101904538, ZSCAN25, TMEM225B, LOC112444278*
25 (194)	36	3.80×10^{-3}	0.98	0.017	*LOC101904538, ZSCAN25, TMEM225B, LOC112444278*
25 (194)	36	3.78×10^{-3}	0.98	0.017	*LOC101904538, ZSCAN25, TMEM225B, LOC112444278*
25 (194)	36	3.76×10^{-3}	0.98	0.017	*LOC101904538, ZSCAN25, TMEM225B, LOC112444278*
25 (194)	36	3.74×10^{-3}	0.98	0.017	*LOC101904538, ZSCAN25, TMEM225B, LOC112444278*
25 (194)	36	3.72×10^{-3}	0.98	0.017	***ZSCAN25,*** *LOC101904538, TMEM225B, LOC112444278, ZNF655*
25 (194)	36	3.70×10^{-3}	0.98	0.017	***TMEM225B, LOC112444278,*** *ZSCAN25, ZNF655, FAM200A, ZNF789*
25 (194)	36	3.68×10^{-3}	0.02	0.017	***TMEM225B, LOC112444278,*** *ZSCAN25, ZNF655, FAM200A, ZNF789*
25 (195)	41	4.51×10^{-3}	0.98	0.017	*ELFN1*
25 (196)	42	4.49×10^{-3}	0.99	0.017	*PDGFA*
26 (197)	7.9	3.37×10^{-3}	0.95	0.012	***PRKG1***
26 (198)	15	3.31×10^{-3}	0.99	0.017	***PLCE1***
26 (198)	15	3.30×10^{-3}	0.99	0.017	***PLCE1***
26 (198)	15	3.29×10^{-3}	0.99	0.017	***PLCE1,*** *NOC3L*
26 (198)	15	3.28×10^{-3}	0.99	0.017	***PLCE1,*** *NOC3L*
26 (198)	15	3.27×10^{-3}	0.99	0.017	***PLCE1,*** *NOC3L*
26 (198)	15	3.26×10^{-3}	0.99	0.017	***PLCE1,*** *NOC3L*
26 (198)	15	3.25×10^{-3}	0.99	0.017	***PLCE1,*** *NOC3L*

26 (198)	15	3.24x10^{-3}	0.99	0.017	***PLCE1,*** *NOC3L*
26 (198)	15	3.23x10^{-3}	0.99	0.017	***PLCE1,*** *NOC3L*
26 (198)	15	3.22x10^{-3}	0.99	0.017	***NOC3L,*** *PLCE1, TBC1D12*
26 (198)	15	3.21x10^{-3}	0.99	0.017	***NOC3L,*** *PLCE1, TBC1D12*
26 (198)	15	3.20x10^{-3}	0.99	0.017	***NOC3L,*** *PLCE1, TBC1D12, LOC112444547*
26 (198)	15	3.19x10^{-3}	0.99	0.017	***NOC3L,*** *PLCE1, TBC1D12, LOC112444547*
26 (198)	15	3.18x10^{-3}	0.99	0.017	***NOC3L,*** *PLCE1, TBC1D12, LOC112444547*
26 (198)	15	3.17x10^{-3}	0.98	0.017	***TBC1D12,*** *NOC3L, LOC112444547*
26 (199)	16	3.16x10^{-3}	0.99	0.017	***CYP2C18,*** *LOC101902226*
26 (199)	16	3.15x10^{-3}	0.99	0.017	***CYP2C18,*** *LOC101902226, LOC112444533*
26 (199)	16	3.14x10^{-3}	0.99	0.017	***CYP2C18,*** *LOC101902226, LOC112444533, CYP2C87*
26 (199)	16	3.13x10^{-3}	0.99	0.017	***LOC112444533,*** *CYP2C18, LOC101902226, CYP2C87*
26 (199)	16	3.12x10^{-3}	0.99	0.017	***LOC112444533,*** *CYP2C18, CYP2C87*
26 (199)	16	3.11x10^{-3}	0.99	0.017	***CYP2C87,*** *LOC112444533, TRNAS-GGA*
26 (199)	16	3.10x10^{-3}	0.99	0.017	***CYP2C87,*** *LOC112444533, TRNAS-GGA*
26 (199)	16	3.09x10^{-3}	0.99	0.017	***CYP2C87,*** *TRNAS-GGA, LOC107131872, LOC112444508*
26 (199)	16	3.08x10^{-3}	0.99	0.017	***LOC540627,*** *LOC100847963*
26 (199)	16	3.07x10^{-3}	0.99	0.017	***LOC100847963,*** *LOC540627*
26 (200)	41	1.32x10^{-3}	0.98	0.014	***FGFR2***
26 (201)	48	5.02x10^{-3}	0.03	0.018	-
26 (202)	48	7.3 x10^{-3}	0.95	0.015	-
26 (202)	48	7.34x10^{-3}	0.95	0.015	-
26 (202)	48	4.97x10^{-3}	0.95	0.018	***MGMT***

26 (202)	48	$4.91x10^{-3}$	0.06	0.018	*MGMT*
26 (202)	48	$4.86x10^{-3}$	0.93	0.018	*MGMT*
26 (202)	48	$4.81x10^{-3}$	0.93	0.018	*MGMT*
26 (202)	48	$4.76x10^{-3}$	0.93	0.018	*MGMT*
26 (202)	48	$4.71x10^{-3}$	0.93	0.018	*MGMT*
26 (202)	48	$4.66x10^{-3}$	0.93	0.018	*MGMT*
26 (202)	48	$4.61x10^{-3}$	0.93	0.018	*MGMT*
26 (202)	48	$4.57x10^{-3}$	0.93	0.018	*MGMT*
26 (202)	49	$4.52x10^{-3}$	0.93	0.018	*MGMT*
26 (203)	50	$6.86x10^{-3}$	0.97	0.015	-
26 (203)	50	$1.21x10^{-2}$	0.95	0.014	*TCERG1L*
26 (203)	50	$3.98x10^{-3}$	0.95	0.019	*TCERG1L*
26 (203)	50	$3.91x10^{-3}$	0.95	0.019	*TCERG1L*
26 (203)	50	$3.85x10^{-3}$	0.95	0.019	*TCERG1L*
26 (203)	50	$3.78x10^{-3}$	0.95	0.019	*TCERG1L*
26 (203)	50	$1.21x10^{-2}$	0.95	0.014	*TCERG1L*
26 (203)	50	$1.21x10^{-2}$	0.95	0.014	*TCERG1L*
26 (203)	50	$1.21x10^{-2}$	0.95	0.014	*TCERG1L*
26 (203)	50	$1.20x10^{-2}$	0.95	0.014	*TCERG1L*
26 (203)	50	$6.89x10^{-3}$	0.95	0.015	*TCERG1L*
26 (203)	50	$3.31x10^{-2}$	0.96	0.013	*TCERG1L*
26 (203)	50	$3.31x10^{-2}$	0.96	0.013	*TCERG1L*
26 (203)	50	$1.20x10^{-2}$	0.95	0.014	*TCERG1L*

26 (203)	50	1.20×10^{-2}	0.95	0.014	***TCERG1L***
26 (203)	50	1.20×10^{-2}	0.95	0.014	***TCERG1L***
26 (203)	50	1.19×10^{-2}	0.95	0.014	***TCERG1L***
26 (203)	51	1.19×10^{-2}	0.95	0.014	*LOC104976020, BNIP3*
26 (203)	51	1.19×10^{-2}	0.95	0.014	-
26 (203)	51	1.19×10^{-2}	0.95	0.014	-
26 (203)	51	1.19×10^{-2}	0.95	0.014	*LOC104975972*
26 (203)	51	1.18×10^{-2}	0.95	0.014	*LOC104975972*
26 (203)	51	3.30×10^{-2}	0.96	0.013	-
27 (204)	13	7.14×10^{-3}	0.98	0.015	***TENM3***
27 (205)	25	4.59×10^{-2}	0.81	0.012	*TRNAE-UUC*
29 (206)	14	1.32×10^{-2}	0.98	0.014	-
29 (206)	14	1.32×10^{-2}	0.99	0.014	*LOC101903773*
29 (207)	15	3.29×10^{-2}	0.96	0.013	-
29 (208)	42	7.33×10^{-3}	0.95	0.015	*NRXN2, LOC112444844, RASGRP2, PYGM*
29 (209)	43	7.32×10^{-3}	0.95	0.015	*SNX15, SAC3D1, NAALADL1, CDCA5, ZFPL1, TMEM262, VPS51*
29 (210)	43	7.30×10^{-3}	0.95	0.015	***POLA2,*** *SLC22A20P, LOC112444880*
X (211)	0.1	4.34×10^{-3}	0.92	0.016	*KLHL4*
X (212)	1.9	4.77×10^{-2}	0.90	0.012	-
X (212)	1.9	4.76×10^{-2}	0.90	0.012	-
X (213)	31	4.64×10^{-2}	0.93	0.012	***AFF2***
X (213)	31	4.63×10^{-2}	0.93	0.012	***AFF2***
X (213)	31	4.63×10^{-2}	0.93	0.012	***AFF2***
X (213)	31	4.62×10^{-2}	0.94	0.012	***AFF2***
X (213)	31	4.62×10^{-2}	0.94	0.012	***AFF2***
X (213)	31	4.61×10^{-2}	0.94	0.012	***AFF2***
X (213)	31	4.60×10^{-2}	0.93	0.012	***AFF2***
X (213)	31	4.60×10^{-2}	0.94	0.012	***AFF2***
X (213)	31	4.59×10^{-2}	0.94	0.012	***AFF2***

X (213)	31	4.59×10^{-2}	0.94	0.012	***AFF2***
X (213)	31	4.58×10^{-2}	0.93	0.012	***AFF2***
X (213)	31	4.58×10^{-2}	0.94	0.012	***AFF2***
X (213)	31	4.57×10^{-2}	0.94	0.012	***AFF2***
X (213)	31	4.57×10^{-2}	0.94	0.012	***AFF2***
X (214)	56	7.76×10^{-3}	0.93	0.015	***COL4A6,*** *LOC112445233*
X (214)	57	4.38×10^{-2}	0.89	0.012	***COL4A5***
X (214)	57	4.37×10^{-2}	0.89	0.012	***COL4A5***
X (215)	80	3.66×10^{-3}	0.98	0.017	***(AWAT1,*** *ARR3, P2RY4*
X (216)	133	7.18×10^{-3}	0.98	0.015	***SHROOM2***
X (216)	133	7.16×10^{-3}	0.98	0.015	***SHROOM2***
X (216)	133	$7.15\ 10^{-3}$	0.98	0.015	***SHROOM2,*** *GPR143*

[1]*Bos taurus* chromosome. In parentheses is the locus associated with spontaneous abortion that is numbered sequentially from chromosome 1 to the X chromosome so that the SNPs that comprise the loci associated with spontaneous abortion may be identified. [2] Location of SNP, in Megabases (Mb), measured by the numbered nucleotides in the ARS-UCD 1.2 reference genome assembly (https://www.ncbi.nlm.nih.gov/datasets/genome/GCF_002263795.1/). [3] FDR corrected *P*-value for each SNP associated with spontaneous abortion. [4] Favorable allele (FA) frequency within the population.
[5] The proportion of variance (POV) explained for each SNP associated with spontaneous abortion.
[6] Positional candidate genes located within 30.5 kb on either side of the SNP(s) associated within each locus. Bolded gene names represent genes where the SNP is located within the gene.

Table S2: Genes associated with spontaneous abortion in heifers bred by artificial insemination that are organized by function

Group definition[1]	No. genes[2]	Genes[3]
Response to stress; conversion of stress hormone cortisol	3	*PIN1, HSD11B1, CDH13*
DNA repair	4	*OOEP, MGMT, CDCA5, KDM5B*
Movement of cilia, located within cilium, regulation of cilium assembly; Cilia movement/Cilium	4	*LOC104968411, ANKS6, IQCD, RABEP2*
Response to inflammation; role in inflammation	5	*PADI4, PAPPA, PTGES, BRINP3, OTULIN*
Reproduction - embryo implantation/oocyte genomic imprinting	5	*KHDC3L, OOEP, CST8, LOC100847115, SPINK2*
Involvement in regulation of translation	5	*RBM47, PPP1R15B, MIR205, DDX24, MIR2284K*
Protein-protein interactions	5	*MPDZ, DCLK1, IFI27, ZSCAN25, ZNF655*
Cytochromes - cholesterol synthesis	5	*CYP3A24, CYP2C18, CYP2C87, LOC100847963, LOC540627*
Transfer RNA's	6	*TRNAC-GCA, TRNAK-UUU, TRNAS-GGA, TRNAS-UCC, TRNAE-UUC, TRNAC-ACA*
GTPase activity/activator	6	*RASA3, ASAP1, RABEP2, TBC1D12, RASGRP2, TBC1D19*
Hormone synthesis	7	*CES5A, CYP3A24, CYP2C18, CYP2C87, LOC100847963, LOC540627, SNX15*
ATPase activity, ATP synthesis	8	*UCP3, UCP2, DNAH5, ATP5MPL, KATNAL2, NUBPL, CYB5R1, RABIF*
Fatty acid & lipid metabolism	9	*UCP3, UCP2, MBOAT7, CYP3A24, CYP2C18, CYP2C87, LOC100847963, LOC540627, AWAT1*
Apoptosis signaling pathway	11	*WDR12, RNAF130, XKR6, G0S2, WWOX, CDH13, TFPT, IFI27, FAM32A, TRIM36, BNIP3*
G Protein-coupled receptors/receptor activity	12	*ADGRL2, ADGRL4, PTGFR, ADRA2C, LOC518134, LOC100337081, LOC532436, OR52H1, MGC137098, LOC101904538, P2RY4, GRK7*
Cellular differentiation involvement	15	*ETV1, RBM47, PIN1, OLFM2, ROS1, OCSTAMP, PTPN14, CDH13, TMEM225B, PLCE1, NOC3L, PRRX1, BCAP29, BMPER, LOC509941*
Membrane structure	15	*LY6L, STUM, LAMB3, MBOAT7, EDDM13, ATP5MPL, PHACTR1, ADTRP, SMIM13,*

		TMEM262, SLC22A20P, COL4A6, CTXN3, ITM2B, COL4A5
Involvement in Immune Response	21	*CCDC80, CD80, MASP1, PADI4, GRAP2, PIN1, PAPPA, CD109, CST11, LOC516599, TRIM5, TRIM34, IL21, BCL11B, SH2B1, CD19, SAC3D1, KLHL12, CMIP, TRIM36, OTULIN*
Calcium binding activity, calcium transport, calmodulin activity, sodium transport, ion transport, Chloride conductance/channel regulation	21	*CLSTN2, SPATA21, CACNA1l, LOC112441544, ANKEF1, SLC9A8, TNR, CAMK1G, TPCN1, VAT1L, TMC4, ZFPL1, NAALADL1, SLC22A0P, EFCAB11, SLC26A4, STIM2, SPINK2, SHROOM2, CLCA1, CLCA2*
Transportation of molecules between cells and within a cell	23	*SIDT1, NECAP2, ADGRL2, HBP1, ENTHD1, NUP50, SLC9A8, GPIHBP1, ASAP1, PIK3C2B, TNR, CES5A, TMC4, TBC1D12, ZFPL1, SLC22A20P, AP1M1, MARCHF3, HEATR5A, STIM2, LOC784385, RABIF, SYT2*
Involved in necessary processes of the cell cycle, DNA replication, mitosis/meiosis	25	*SPICE1, WDR12, CROCC, OOEP, EHMT1, PIGU, PTPN14, CCNK, CEP192, KATNAL2, SH2B1, TMEM225B, NOC3L, CDCA5, SAC3D1, POLA2, ARR3, RNF7, LOC101904827, RAD51B, MTUS2, GPC6, GORAB, LOC784385, CENPN*
Involvement in Metabolic pathways - protein, carbohydrates, steroid	26	*CFAP44, TMRPSS2, SLC2A10, PAAF1, COP1, CLEC3A, IFI27, HHIPL1, ADTRP, CEP192, TMEM225B, ELFN1, GCH1, FUT8, SPON1, FMO3, GAN, NUBPL, MRPS21, APH1A, CA14, TBC1D19, GALNT12, CYB5R1, NAALADL1, WWOX*
Nervous system - voltage gated channels, neurotransmitter release, neuronal responses, function as receptors in nervous system	25	*CLSTN2, CNTNAP2, CACNA1l, LOC112441544, EPHA5, ADRA2C, KCNQ5, GRIK2, DCLK1, NBEA, LOC532436, OR52H1, MGC137098, CDH13, LOC101904538, ELFN1, TENM3, NRXN2, TNR, DPYSL2, GAN, REST, CAMSAP2, SYT2, MAP1B*
Involved in signal transduction and signaling pathways	30	*DGKB, ARRDC1, RASA3, SPATA2, SLC9A8, RHPN1, ZC3H3, LY6H, PIK3C2B, LRRN2, ITPKB, HHAT, LOC112442091, TFPT, TMEM170B, SMAD4, SH2B1, PRKG1, PLCE1, GPR143, WDR12, RNF7, NRCAM, WDHD1, KLHL12, AKAP6, AMER3, TRIM36, ADIPOR1, OTULIN*

Regulation of alternative splicing/small nucleolar RNA - critical components of the spliceosome	31	*LOC112446095, LOC100297616, LOC112446494, LOC112447606, LOC112447604, LOC112449153, LOC112449430, LOC101905203, LOC112449409, LOC112441704, LOC112441896, LSM14A, PRPF31, DDX24, LOC112444235, LOC112444219, TMEM225B, LOC112444547, LOC112445233, RBFOX1, LOC112448292, LOC112448294, LOC112447194, LOC112447625, LOC112447896, LOC112449404, LOC112449405, LOC112449403, LOC112443378, LOC112443346, LOC51519*
Transcription factor activity; transcription factor; regulation RNA polymerase II regulation; transcription coregulatory activity; regulation of transcription	42	*CARF, RPRD2, ETV1, HBP1, SOX5, HMX1, OOEP, EHMT1, TP53INP2, EYA2, ZMYND8, SMYD2, MIR205, MAF, CDYL2, ZNF583, ZNF667, IFI27, BCL11B, CCNK, MIR2284K, LOC104975783, SMAD4, TMEM225B, ZSCAN25, ZNF655, ZNF789, NOC3L, TCERG1L, YLPM1, SERTAD2, PRRX1, KDM5B, TCF4, AFF2, SAMD7, CIART, REST, ZNFX1, ZNF281, ZNF354C, ZNF879*
Involved in processes related to development of embryo, fetus and cells	49	*UPK1B, PADI6, ECM1, HS2ST1, ETV1, ACTR3B, SOX5, EPHA5, RNF130, PIN1, XKR6, ANKS6, PAPPA, OOEP, ROS1, STARD13, CDH22, EYA2, PIK3C2B, TNR, PTPN14, HHAT, CTNND2, DDX24, CCDC85C, SH2B1, RABEP2, PDGFA, PRKG1, PLCE1, NRXN2, SNX15, NRCAM, LOC101904827, DPYSL2, SERTAD2, MTUS2, GPC6, SPON1, PRRX1, EGFR, TCF4, FGFR2, BMPER, LOC509941, TRIM36, LOC784385, CAMSAP2, MAP1B*
Other	126	*LOC112447301, B4GALT4, TIMMDC1, LOC112447304, ADPRH, PLA1A, LRRC31, LOC100300383, RTP1, LOC783376, TARS2, LOC101903905, LOC112446000, LOC112445906, LOC112446001, LOC112445881, LOC104971683, LOC101907567, COG5, CPSF6, LOC101901948, LOC782958, LOC112447356, LOC104969267, LOC112448016, LOC112448015, LOC104972957, DDX43, LOC104972961,*

		DPH7, SMYND19, WDFY2, FREM2, LOC107132979, LOC112449139, LOC112449104, C12H13orf46, LOC104973683, LOC531692, MGC133636, CSTL1, LOC617402, LOC100295994, LOC786401, LOC101904842, LOC104973899, LOC112449339, LOC104973934, LOC112449243, MAFA, PSKH2, LOC104974137, COL14A1, LOC107131357, LOC788136, LOC101902757, LOC112441550, LOC112441809, LOC101907913, MDM4, LOC100335838, LOC101902053, LOC100848409, LOC112441869, LOC104974530, LOC104974529, LOC112442028, LOC112442252, LOC101902700, LOC101905303, LENG1, LOC101905919, FBXL7, ANKH, OTULINL, LOC107131599, ISG12(B), LOC514011, RD3L, LOC112443493, LOC104976682, LOC104969833, LOC112444168, LOC107131825, LOC101908406, LOC112444278, FAM200A, LOC112444533, LOC101902226, LOC112444508, LOC107131872, LOC112444844, VPS51, LOC112444880, KLHL4, LOC104968752, LOC112446461, LOC107132618, LOC112447389, LOC112447394, LOC787395, MROH9, LOC112441831, LOC112441775, LOC101902820, LOC101908185, C3H1orf54, LOC112446328, LOC112447154, LOC783045, LOC783735, LOC101907299, UBL3, LOC112449055, LOC100847759, LOC101901943, LOC517828, LOC112441773, LOC100337507, CMC2, LOC107131620, LOC112443476, LOC112444294, LOC104976020, LOC104975972, LOC101903773

[1]Description of shared function of genes in each group. [2] Number of genes in each group. [3] List of genes in each group.

Table S3: Relative risk for fetal loss with the undesirable allele for the most significant single nucleotide polymorphisms associated with fetal loss for 216 loci.

BTA[1]	Position (Mb)[2]	Locus No.[3]	Relative Risk[4]
1	58.1	1	3.30
1	58.2	2	3.90
1	62	3	4.75
1	63	4	3.12
1	64	5	2.13
1	64.3	6	3.27
1	76	7	2.19
1	80	8	1.18
1	97	9	5.49
1	127	10	2.40
1	128	11	1.38
1	141.7	12	4.10
1	141.8	13	3.85
1	146	14	1.59
2	1.4	15	3.38
2	15	16	2.54
2	91	17	1.51
2	109	18	3.16
2	123	19	1.66
2	135	20	2.05
3	20	21	2.33
3	57	22	2.20
3	62	23	2.31
3	63	24	2.31
3	65	25	1.91
3	66	26	1.26
3	82	27	1.91
4	19	28	1.96
4	21	29	1.16
4	22.4	30	1.48
4	22.9	31	1.74
4	30	32	1.17
4	48	33	1.43
4	49	34	1.61
4	52	35	1.04
4	62	36	1.31
4	111	37	2.90
4	115.1	38	2.94
4	115.4	39	1.77
4	117	40	1.38
5	11	41	1.70

5	13.1	42	2.52
5	13.3	43	1.79
5	20	44	1.64
5	44	45	1.97
5	85	46	1.72
5	111	47	2.39
5	115	48	1.79
6	15	49	1.80
6	43	50	1.60
6	45	51	3.16
6	46	52	1.86
6	46.2	53	1.56
6	54	54	2.62
6	59	55	2.42
6	71	56	2.25
6	81	57	2.54
6	86	58	1.05
6	115	59	1.91
7	1.1	60	1.74
7	2.3	61	1.37
7	6.8	62	2.24
7	14	63	2.12
7	17	64	1.51
7	26	65	2.09
7	29	66	1.44
7	32	67	3.04
7	89	68	1.37
7	103	69	1.36
8	8.2	70	2.45
8	11	71	3.20
8	31	72	1.71
8	35	73	1.82
8	63	74	1.78
8	73	75	1.70
8	97.1	76	1.06
8	97.8	77	1.08
8	97.9	78	1.42
8	105.3	79	1.28
8	105.5	80	1.21
9	7.8	81	2.63
9	12	82	1.83
9	12.8	83	1.84
9	13	84	2.69
9	33	85	1.48
9	48	86	1.70
10	3.9	87	1.34

10	44	88	1.51
10	67	89	1.09
10	77	90	3.21
10	80	91	2.47
10	86	92	1.07
10	99	93	1.51
10	101	94	1.13
11	63	95	2.12
11	100	96	1.08
11	106	97	1.37
12	18	98	1.98
12	21	99	2.54
12	23	100	1.92
12	25	101	1.95
12	27	102	1.73
12	31	103	1.97
12	53	104	1.83
12	56	105	2.78
12	68	106	1.68
12	79	107	2.35
12	86	108	1.13
13	3.2	109	1.57
13	5.5	110	1.62
13	41	111	1.09
13	42	112	1.04
13	64	113	1.37
13	75.6	114	1.07
13	75.7	115	2.22
13	77	116	2.02
13	79	117	1.36
14	1.3	118	1.29
14	10	119	2.74
14	76.8	120	1.67
14	76.9	121	4.32
14	81	122	1.30
15	15	123	1.04
15	25	124	1.57
15	32	125	1.40
15	38	126	1.15
15	48	127	1.79
15	53	128	2.42
16	2.3	129	1.64
16	15	130	1.46
16	29	131	1.25
16	38	132	1.28
16	40	133	1.23

16	56	134	1.24
16	57	135	1.08
16	69	136	1.50
16	73	137	2.96
16	73.6	138	1.62
16	73.8	139	1.51
16	78	140	1.45
16	80	141	1.70
17	14.8	142	2.97
17	14.9	143	2.83
17	15	144	2.97
17	35	145	2.42
17	61	146	1.50
17	64	147	1.53
18	5.1	148	1.94
18	5.5	149	1.09
18	5.6	150	1.83
18	5.8	151	1.20
18	6.4	152	2.07
18	6.5	153	2.39
18	7	154	2.20
18	7.1	155	2.26
18	7.6	156	2.93
18	7.9	157	1.65
18	9.3	158	1.50
18	24	159	1.67
18	41	160	2.29
18	44	161	4.10
18	63.2	162	1.89
18	63.7	163	3.12
20	9.5	164	1.31
20	57.5	165	1.30
20	57.6	166	1.29
20	58.3	167	1.40
20	58.6	168	1.39
20	60	169	1.69
20	61	170	2.15
20	68	171	2.30
21	21	172	1.81
21	42	173	1.74
21	58	174	2.71
21	64.1	175	1.66
21	64.6	176	1.75
21	68	177	1.81
22	1	178	1.17
22	3.5	179	1.30

22	5.5	180	1.22
22	30	181	1.34
22	31	182	1.16
23	43	183	1.43
23	44	184	1.91
23	45	185	1.83
24	1.8	186	1.58
24	43	187	1.52
24	46	188	1.70
24	50	189	1.71
24	51	190	1.82
24	54	191	2.13
25	6.2	192	1.07
25	25	193	1.37
25	36	194	1.03
25	41	195	2.07
25	42	196	2.17
26	7.9	197	1.66
26	15	198	1.51
26	16	199	1.46
26	41	200	2.71
26	48	201	1.67
26	49	202	1.18
26	50	203	1.77
27	13	204	2.03
27	25	205	1.66
29	14	206	3.32
29	15	207	2.38
29	42	208	1.16
29	43.2	209	1.40
29	43.4	210	1.35
X	0.1	211	1.90
X	1.9	212	1.68
X	31	213	1.28
X	56	214	1.37
X	80	215	1.75
X	133	216	1.09

[1]*Bos taurus* chromosome where SNP is located. [2]Location of SNP, in Megabases (Mb), measured by the numbered nucleotides in the ARS-UCD 1.2 reference genome assembly (https://www.ncbi.nlm.nih.gov/datasets/genome/GCF_002263795.1/). [3]The locus associated with spontaneous abortion that is numbered sequentially from chromosome 1 to the X chromosome so that the SNPs that comprise the loci associated with spontaneous abortion may be identified. [4]The calculated relative risk for the most significant SNP representing a locus.
